# Evaluating novel and conventional cell‐separation techniques for sexual assault investigations

**DOI:** 10.1111/1556-4029.70131

**Published:** 2025-07-11

**Authors:** Janine Schulte, Simon Egger, Sarah Kron, Eva Scheurer, Iris Schulz

**Affiliations:** ^1^ Institute of Forensic Medicine University Basel Basel Switzerland

**Keywords:** cell‐separation methods, DEPArray™, differential extraction, laser capture microdissection, prostate‐specific antigen, sexual assaults

## Abstract

Biological evidence from sexual assaults frequently includes few male cells (i.e., spermatozoa) and numerous female cells (i.e., epithelial cells). In practice, their genetic analysis typically involves separating the victim's cells from the perpetrator's sperm using conventional differential extraction or advanced cell enrichment/capturing techniques. A descriptive study on simulated sexual assault samples was carried out by the recruitment of 10 heterosexual, monogamous couples. Post‐coital swabs were collected before and after consensual sexual intercourse, with a sampling period of up to 96 h, and subjected to analysis to detect, quantify, and genotype adhering sperm by three distinct cell‐separation techniques: differential extraction, laser capture microdissection, and DEPArray™. Methods differed in sperm detection and genotyping efficacy, while foreign DNA was identifiable up to 96 h. Time since intercourse and individuals were statistically significant factors (*p* ≤ 0.05) on male DNA yields, while hygienic behavior was not. Prior sperm enrichment was pivotal for cell capture technologies to counteract the abundance of epithelial cells, achieved by a prior mild digestion step for laser microdissection. Evaluating the advantages and disadvantages of standard and advanced methods provided a novel, comprehensive understanding of their merits, postulating that modern applications can assist conventional ones in challenging crime samples.

**Highlights**

- Cell‐separation methods (DE, LCM, and DEPArray™) were applied on post‐coital samples.
- Sperm and DNA from the minor contributor were identifiable up to 96 h.
- Cell‐separation methods differed in their sperm detection and genotyping efficacy.
- Time since intercourse and individuals were statistically significant factors on male DNA yields.
- Prior sperm enrichment is desirable for cell‐capturing technologies.

## 1 INTRODUCTION

Gynecological swabs from medico‐legal examinations frequently disclose biological mixtures consisting of minimal quantities of the perpetrator's semen and, in contrast, substantial non‐semen bodily fluids (e.g., saliva, vaginal secretions, and/or blood) predominantly from the victim. Given the frequently limited male contribution, it is pivotal to enrich sperm to genetically identify suspects in sexual assault case mixtures, for example, by differential extraction (DE) [1, 2, 3]. In brief, DE uses a first mild lysis step to disrupt non‐sperm cells (e.g., epithelial cells), followed by centrifugation to separate the still‐intact sperm from the supernatant (non‐sperm fraction (NF)). After transferring the NF to a new collection tube, the sperm‐containing pellet is lysed with the aid of detergents (e.g., SDS) and the reducing agent, DTT, yielding the sperm fraction (SF). Both fractions undergo independent processing for STR genotyping, with the NF ideally containing negligible or no sperm DNA, whereas the SF should be strongly enriched with sperm DNA [3, 4, 5]. In cases where a limited number of sperm cells are anticipated, such as those with delayed reports, high levels of post‐offense activities, or insufficient initial material from the suspect, the enrichment of sperm cells becomes essential. According to the WHO [6], the “optimal” window for collecting forensic evidence is supposed to be within 72 h post‐assault, with immediate collection being particularly beneficial [7]. However, many jurisdictions do not strictly adhere to a 72‐h sampling cutoff, with time limits varying from 24 to 48 h or contingent upon various factors such as the victim's age or the context of the crime [8].

Even though DE is the gold‐standard method in forensic DNA laboratories due to its simplicity, high efficiency, and low costs, it faces some drawbacks and limitations. For instance, separation is still influenced by possible carryover between the fractions, meaning that separation into two distinct cell populations is not consistently successful. Notably, male material within the NF could be the result of (1) premature lysed sperm, (2) male epithelial cells, or (3) pipetting inaccuracies. In contrast, female DNA carryover might be found in the SF due to (1) insufficient mild lysis or (2) the inadequate removal of the supernatant after mild lysis [9]. Moreover, the DE process exclusively enables the separation of sperm and non‐sperm cells; thus, the same cell type of different contributors cannot be differentiated, thereby confining its applicability solely to sperm‐containing samples. Lastly, scientific research has supposed that the process of DE necessitates a minimum quantity of sperm at the outset to achieve the offender's genotype [10, 11]. Some material loss, however, will inevitably occur during the transfer, pipetting, extraction, and purification process [11, 12]. Hence, since its first report in 1985 [3], there have been continued efforts to optimize DE, for example, by adapting washing steps [5], using filtration methods [2], or increasing automation [13, 14, 15].

Next to DE, several applications have emerged that allow the physical separation of forensic mixtures into (1) sub‐populations or (2) individual cellular types prior to genetic analysis. For instance, fluorescence‐activated cell sorting (FACS), as well as magnetic‐activated cell sorting (MACS), were found suitable for separating sub‐populations of epithelial, sperm, and blood cells based on tissue‐specific and chromosome‐specific features (FACS) or cell‐specific antigens utilizing immune‐magnetic beads, which are usually coupled with antibodies (MACS) [16, 17]. Importantly, though, large initial sample volumes and imperfect separation due to fluorescent crosstalk between populations have been reported for FACS analysis [16, 18], while the manipulation of semen stains for the MACS method has been associated with increased sample loss [17]. Again, if a mixed sample comprises donors from the same cell type, separating them into single‐source cell populations is generally challenging [16].

In contrast, cell capture strategies (also known as single‐cell technologies) allow for genetic analysis at the single‐cell level so that each cellular fraction or individual cell can be assigned to a specific cell type and thus potentially a contributor. Single‐cell separation enables the deconvolution of complex mixtures with more than two contributors or homogenous (same‐cell) mixtures but is associated with the challenge of successfully genotyping the tiny DNA quantities. Usually, this approach is linked to heightened stochastic effects (e.g., peak imbalance, increased stutter frequencies and heights, and drop‐ins) and incomplete profiling (i.e., dropouts). Thus, adjustments to the lab workflow (e.g., PCR cycles, PCR volumes, and threshold settings) have already been introduced for single‐cell analysis [16, 19, 20].

One inexpensive cell separation technique is micromanipulation, enabling image‐based detection and precise physical control and manipulation of target cells. Although some manual dexterity is required depending on the method (e.g., manual approaches with micro‐/pico‐pipettes, tungsten needles, micromanipulators, and optical tweezers), micromanipulation has already proven successful in forensic buccal and sperm cell studies [16, 21, 22, 23]. Laser microdissection (LMD), an image‐based technique, couples light microscopy with UV or IR laser beam technology that allows for the specific separation of cell and tissue regions of interest [17, 24, 25]. In the forensic context, LMD is often used interchangeably with laser capture microdissection (LCM) and is here further referred to as such. While LCM has been proven effective in isolating male genetic profiles from recovered sperm cells, it is important to note that in recent studies, a minimum of 30–150 pooled sperm cells was necessary to obtain complete male profiles [24, 26], with sperm retrieval being a time‐intensive endeavor [27, 28]. Additionally, the utilization of a laser beam could cause DNA damage.

Another image‐based cell targeting technique was introduced with the DEPArray™, a semi‐automated digital cell sorter. With this technique, fluorescent‐tagged epithelial, blood, or sperm cells in every conceivable mixture constellation are detected and identified under image‐assisted technology and recovered in a controlled manner using di‐electrophoresis [29]. The applied electro‐kinetic principle has been shown to be gentle, not affecting the cells' DNA quality and thus the genotyping results, providing improved results compared to dilution series [19] while only requiring approximately 15–20 single sperm for consensus profiles and 20–30 simultaneously recovered sperm to obtain pooled profiles [29]. Nevertheless, the utilization of the technology poses challenges in terms of fiber particles, rendering the method less suited for evidence not collected with nylon swabs [18, 29]. In addition, the ability to process the entire sample is limited due to the cell capacity of the DEPArray™ cartridge, while the evaluated cells may not be accessible owing to their condition or trapping location [29, 30].

The conventional protocol for addressing sexual offenses in our laboratory involves presumptive testing for body fluid identification using immunoassays (i.e., assessment of prostate‐specific antigen (PSA) levels) and microscopic confirmation of sperm cells. If positive, the samples are subjected to DE or, otherwise, regularly extracted, quantified, and genotyped following internally established and validated protocols according to ISO/IEC 17025‐accredited laboratory standards. Thus, the preliminary PSA and microscopy results determine whether DE is performed in the routine downstream workflow of sexual assault samples. As two cell‐capture techniques are available on‐site, this study presents a novel comparison of the three forensic mixture deconvolution techniques, (1) DE (conventional), (2) LCM, and (3) DEPArray™, on sperm detection and genotyping. The evaluation is conducted using post‐coital swabs sampled over 4 days to mirror gradually declining sperm counts, as often encountered with delayed reports of victims. We propose that cell‐based targeting technologies might enable enhancements to the standard workflow for forensic DNA analytical laboratories, which include (1) simultaneous detection, identification, and isolation of target cells (e.g., sperm) based on morphological properties, staining prior to DNA extraction, and respective recovery mechanisms, (2) improvements in the sensitivity of sperm detection, particularly in samples with few sperm present, and (3) increased mixture resolution and interpretability of low‐template DNA samples. Finally, the study provides essential information about the advantages, disadvantages, and technical data of the respective methods as well as the applicability, sensitivity, and performance with regard to the processing of vaginal smears up to 96 h after physical contact.

## 2 MATERIALS AND METHODS

### 2.1 Study design, recruitment, and procedure

A monocentric prospective and descriptive study with risk category A was conducted and approved by the local ethics committee (BID 2021‐02097). All procedures performed in studies involving human participants were in accordance with the ethical standards of the institutional and/or national research committee and with the 1964 Helsinki Declaration and its later amendments or comparable ethical standards [31, 32, 33]. Written informed consent was obtained from all participants.

After unprotected, consensual sexual intercourse with intravaginal ejaculation, 10 monogamous, heterosexual couples (referred to as the study cohort) were asked to perform post‐coital intravaginal sampling using CE‐certified nylon‐flocked swabs (ForensiX Evidence Collection Kit, Prionics AG, Schlieren, Switzerland) as recommended or applied in previous studies [9, 18, 29, 34, 35]. Samples were taken as follows: before consensual intercourse (baseline sample), immediately after intercourse (T0; T = time), and at time points T12 to T96 at 12‐h intervals, resulting in nine specific time points after sexual intercourse, respectively. The post‐coital sampling time point is referred to as the time since intercourse (TSI). Excessive deviations (≥1 h) from the scheduled sampling time points led to the exclusion of the corresponding time point from the data analysis. Participants had to refrain from sexual intercourse for 1 week to prevent any (male) DNA material residues. Females were required to wait at least 5 days post‐menstruation before sampling commenced to avoid the presence of menstrual blood residues. The timings of sample collection before and after sexual intercourse, along with shower/bath times, contraceptive methods employed, the date of the last and next menstruation, and the application of vaginal douches and lubricants were recorded. Two buccal swabs per person were taken and analyzed to create reference profiles.

At each time point, three swabs (labeled 1–3) were used sequentially to swab the vaginal cavity for a maximum of 5 s each. The swabs were applied in a couple‐specific, randomized order (Table S1) to minimize sampling bias. One swab was then processed for one method, that is, DE (including PSA testing and microscopy), LCM, and DEPArray™. In total, 300 swabs consisting of 20 reference samples, 10 baseline samples, and 270 study samples were analyzed. Baseline samples were initially designated for the detection of residual PSA. However, as 5/10 baseline samples showed positive PSA tests (Figure S1), microscopy and DE were performed to ensure that participants had adhered to abstinence.

### 2.2 Sample storage until processing

Samples were returned no later than 7 days after sampling started, and stored at 4°C upon arrival to prevent potential microbial growth until processing [9]. Cellular material was released from swab heads and brought into solution according to the method‐specific procedures described below. In the case of LCM, microscopic examination was performed directly after HE staining since previous studies have shown that the staining reagents and storage can interfere with DNA profiling of microdissected cells [24, 26]. The fluorescently labeled cell suspensions for the DEPArray™ analysis were also processed immediately.

### 2.3 Standard workflow—Detection of PSA and spermatozoa

For baseline (*n* = 10) and DE‐processed study samples (*n* = 90), PSA testing was performed using PSA Semiquant CS kit (Seratec® GmbH, Gottingen, Germany) according to the manufacturer's protocol [36] except for a prolonged incubation time of 2 h in 250 μL PSA buffer at room temperature according to our internal validated protocol. Results were documented 10 min after application, and outcomes were considered positive (test result line “T‐line” clearly visible along with the control line), weakly positive (“T‐line” faintly visible along with the control line), or negative (“T‐line” absent).

For spermatozoa detection, 2 μL of the cell pellet from the PSA workflow was pipetted onto an ethanol‐decontaminated microscope slide. The sample space on the slides was previously marked with a peroxidase‐anti‐peroxidase pen (PAP pen, Sigma‐Aldrich, St. Louis, USA). After drying for at least 2 h, the samples were dyed with Hemacolor® rapid staining set (Merck Millipore, Burlington, USA) according to the manufacturer's recommendation, including a positive control per batch with a diluted seminal fluid sample (2 μL, 1:500). Spermatozoa identification and counting were conducted within the pipetted 2 μL spot using the Zeiss Axioskop 40, Zeiss AxioObserver Z1 (Carl Zeiss AG, Oberkochen, Germany) microscope. Spermatozoa were counted up to a maximum of 10 cells, even if more were present, as the aim was to report the presence or absence of spermatozoa.

### 2.4 Standard workflow—Cell separation by differential extraction

DE was performed using the Erase Sperm Isolation Kit (PTC Laboratories, Columbia, USA) according to the manufacturer's protocol [37]. Samples (including cell fractions and reference samples) were extracted on a Maxwell® RSC Instrument (Promega Corporation, Madison, USA) using the Maxwell® FSC DNA IQ™ Casework Kit (Promega) [38], with a final elution volume of 50 μL.

### 2.5 Standard workflow—DNA quantification

Autosomal and Y chromosomal DNA concentrations of sperm and epithelial cell fractions were estimated by real‐time polymerase chain reaction (PCR). The PowerQuant® kit (Promega) was used according to the manufacturer's protocol [39] but in half‐reaction volume according to internal validation on a 7500 PCR Real‐Time System (Applied Biosystems, Foster City, USA) in triplicate and evaluated using PowerQuant® software v. 4.8.0.0 (Promega).

### 2.6 Cell detection and capturing workflow—Cell separation by laser microdissection

Cellular material (*n* = 90) was released from the swab heads using 250 μL PSA buffer from the PSA Semiquant CS Kit (Seratec® GmbH) as described above. The cell suspension was scratched out on a PEN (Polyethylene naphthalate) membrane slide (Carl Zeiss Microscopy), as it stabilizes the sample and is highly absorptive in the UV‐A range, facilitating laser cutting. Two different approaches were applied for the LCM analysis. On one slide, one part of the cell suspension (~2–4 μL) was spread widely using a pipette tip to avoid epithelial cell overlap. With the other part of the same cell suspension, mild digestion of non‐sperm cells was performed and applied on a second slide (as described above) to enrich the spermatozoa due to the low sperm counts, excessive epithelial cells, and the intense time required to screen an entire slide. Samples disposed of on slides were air‐dried for at least 2 h and HE‐stained as described above.

All experiments were performed with the PALM MicroBeam laser microdissection system (PALM Microlaser Technologies, Bernried, Germany), including an inverse microscope AxioObserver Z1 (Carl Zeiss Microscopy) at 400‐fold magnification. Desired cells were cut out and collected by laser pressure catapulting using a UV laser (wavelength 337 nm) operated by the PALMRobo Software (Version 4.3 SP2). Cells were directly catapulted into the cap of an opaque coated 0.5 mL tube (AdhesiveCap 500, Carl Zeiss Microscopy). A minimum distance between the slide and collection cap was chosen, that is, there was no visible gap between the cap and slide. Recovery of the isolated cells in the collection cap was verified microscopically by using the “Cap Check” function of the PALMRobo‐Software. Each slide was manually searched for sperm, with a maximum of 50 cells simultaneously isolated (i.e., pooled). Preliminary experiments and other studies have reported that approximately 150 pg of DNA [19] or 50 sperm [40] can be sufficient for autosomal short tandem repeat (STR) profiling. Microdissected cells were lysed in the same tube using 15 μL buffer from the SwabSolution™ Kit (Promega) containing 1 μL 1:4 diluted 1‐thioglycerol (Maxwell® FSC DNA IQ™ Casework Kit (Promega)) at 70°C for 4 h in an incubator (Labnet International Inc., Edison, USA) according to our internal validated protocol.

### 2.7 Cell detection and capturing workflow—Cell separation by DEPArray™

Samples were processed into an immunofluorescently labeled cell suspension using the DEPArray™ Forensic Sample Prep Kit (Menarini Silicon Biosystems, Bologna, Italy) according to the manufacturer's protocol, except for an adjusted swab suspension volume of 500 μL autoMACS® Running Buffer (Miltenyi Biotec, Bergisch Gladbach, Germany) and incubation at 300–500 rpm for 30 min at room temperature. After centrifugation, the supernatant was removed, and approximately 1 μL of 20 μL of cell suspension was used for automated cell counting using the Countess 3 (Thermo Fisher Scientific) prior to staining approximately 10,000 cells. The stained cell suspensions were counted a second time using the Countess 3 (Thermo Fisher Scientific), and a maximum of 6000 cells were loaded into DEPArray™ cartridges and processed according to the specifications for the DEPArray™ PLUS system (forensic setting). According to the manufacturer's verbal statement, the Countess 3 is able to count cells with a size range of 4–60 μm and concentrations in the range of 1 × 10^4^ to 1 × 10^7^ cells/mL.

Only the epithelial (Fluorescein isothiocyanate (FITC)‐coupled) and sperm cell targeting (allophycocyanin (APC)‐coupled) antibody was used for staining because limited amounts of white blood cells were expected due to the study design, and staining artifacts were reduced (i.e., background, false positives, and spectral overlap). Cell selection was performed via the Cell Browser™ (Menarini Silicon Biosystems, software version 4.0.1.11.3) by evaluating cell routing properties, cell morphology, cell position, and fluorescence signal intensity. For this purpose, only routable cells were screened that are located in a chip cage of the DEPArray™ and can be collected. Conversely, non‐routable cells are positioned in areas of the chip that do not facilitate collection. This may include cells lacking a direct pathway to the parking chamber, likely due to surrounding obstacles, or cells close to the edge of the cartridge. Sperm cells and sperm co‐located with epithelial cells were collected and simultaneously isolated (i.e., pooled) within one reaction tube for subsequent genetic analysis, further designated as recovery. The number of cells per pool and the total number of recoveries were decided case by case based on the amount and condition of detected sperm and hence differed between samples. Single spermatozoa were simultaneously recovered when feasible. In the case of sperm‐epithelial clusters, precautions were taken to maintain a cell type ratio not exceeding 1:10 to identify the minor contributor [11]. Volume reduction was performed according to the manufacturer's recommendations using the VRNxT instrument (Menarini Silicon Biosystems) to avoid cell loss and ensure an equal final volume of 2 μL in each tube for DNA extraction, thereby mitigating user‐specific variable results [19, 41]. Cells were lysed, and DNA was extracted using the DEPArray™ LysePrep Kit (Menarini Silicon Biosystems) according to the manufacturer's instructions.

### 2.8 STR typing and CE

STR typing was performed using the AmpFℓSTR® NGM Detect™ kit (Thermo Fisher Scientific) according to the manufacturer's recommendations on a Veriti™ 96‐Well thermal cycler (Applied Biosystems) with 30 PCR cycles. For DE‐processed samples, if applicable, a total amount of 0.5 ng of sample DNA with a maximum of 15 μL input volume was added to PCR in full reaction volume (25 μL). In the case of DEPArray™ and LCM analysis, the multiplex PCR mix was directly added to the tube. Here, microdissected cells were amplified in full reaction volume, while DEPArray™ recovered cells were amplified in half‐reaction volume (12.5 μL), following the in‐house validation protocol and based on prior published studies [19, 20, 29, 30]. Full‐ or half‐reaction volumes should not affect mean profile completeness while increasing peak heights, as shown in [19] for DNA dilutions and DEPArray™‐recovered single cells. Amplified samples were run on an ABI Prism 3500xL Genetic Analyzer (Applied Biosystems, run voltage 15 kV, injection voltage 1.2 kV, injection time 24 s). Data output was processed using GeneMapper ID‐X v.1.6 Software (Applied Biosystems) with an analytical threshold of 50 RFUs [19, 29].

### 2.9 Profile quality parameter

An STR profile was considered complete when it included all 16 autosomal loci of the used STR kit, including the European Standard Set FGA, TH01, VWA, D1S1656, D2S441, D3S1358, D8S1179, D10S1248, D12S391, D18S51, D21S11, D22S1045, the additional loci D2S1338, D16S539, D19S433, and SE33. Since Amelogenin is not included in the database criteria, it was not considered for profile interpretation [42]. Total allele counts and shared alleles obtained from the reference samples per participant are depicted in Table S2.

Profile completeness was determined by counting the number of donors' alleles present compared to their expected reference profiles. Partial profiles were defined as profiles that contained at least four STR loci. With more than three loci showing alleles that do not originate from the female and male donors, the corresponding DNA profile was classified as a DNA mixture, as described by Hansson et al. [43], and otherwise assigned as a profile from a single source. Only the male component was analyzed as a subject of interest. Mixed profiles were categorized as follows: (1) male profile in the main component and (2) male profile in the minor component. The latter was further defined as (1) not interpretable with <8 alleles (excluding stutter peaks and shared alleles), (2) comparable with ≥8 alleles (excluding stutter peaks and shared alleles), and (3) interpretable or (nearly) complete profiles, allowing a maximum of 4 allelic dropouts. Profiles obtained from the different DEPArray™ recoveries were combined into a composite profile by merging DNA profiling information from replicate profiles derived from the same DEPArray™ run.

### 2.10 Statistical and data analysis

Descriptive statistical analysis and data visualization were performed using R statistical software [44].

To test the hypothesis that time significantly affects male DNA quantity, a linear mixed‐effects model (random intercepts) was estimated, using TSI as a fixed effect and participants as a random effect. A Hausman test compared the random effects model with a fixed effects model for participant study ID. The test did not show any significance (*p* = 0.8911), indicating that a random effects model is better suited for estimation than a fixed effects model. In addition, the model's goodness of fit with random effects was compared to a model without random effects (nor fixed effects) for participants using a likelihood‐ratio test. The test showed high significance (*p* < 0.001), highlighting the importance of random effects for participant study ID.

Regression assumptions were checked by diagnostic plots. When plotting the relationship between male DNA yield (ng/μL) and TSI (Figure S2), it was evident that there was a more likely exponential decrease in DNA quantity during the initial hours. This trend was further supported by the model fit when using a non‐transformed value for DNA quantity. Thus, log‐transformed DNA quantity was used as a dependent variable in the mixed model to better evaluate the average DNA decrease per 12‐h time point.

To statistically evaluate the impact of showering on DNA yields, the relative differences in measured DNA quantity were calculated between each adjacent time point for each participant. This resulted in eight time‐point differences for each couple. A dummy variable was created to indicate whether the participant had showered during each of the calculated time windows. A linear mixed model was then estimated, using the shower‐dummy as an independent variable and the relative differences between time points as the dependent variable, controlling also for time point and using random intercepts for participant study ID.

For DEPArray™ analysis, estimating cell counts after removing cells from the carrier material (i.e., nylon swabs) is desirable to stain approximately 10,000 cells and load 3000–6000 cells. For cell count comparison, the Kruskal–Wallis test with Dunn's post hoc test for multiple, pairwise comparisons was applied (*n* = 90). Data distribution was previously evaluated with the Shapiro–Wilk normality test, density, and Q–Q plots using the dplyr and ggpubr packages [45, 46]. Since not all loaded cells are “routable,” the proportion (relative frequency) of non‐routability was calculated in relation to the total events. The potential association between events and overall non‐routability was assessed by Pearson correlation and Spearman's rank order coefficient, as data appeared not to be strictly linear or normally distributed.

Statistical tests were performed two‐sided, with a significance level of 95% or an alpha of 0.05. Data visualization was conducted using the ggplot2 package [47].

## 3 RESULTS

### 3.1 Standard workflow

#### 3.1.1 PSA testing and sperm cell detection

**FIGURE 1:**
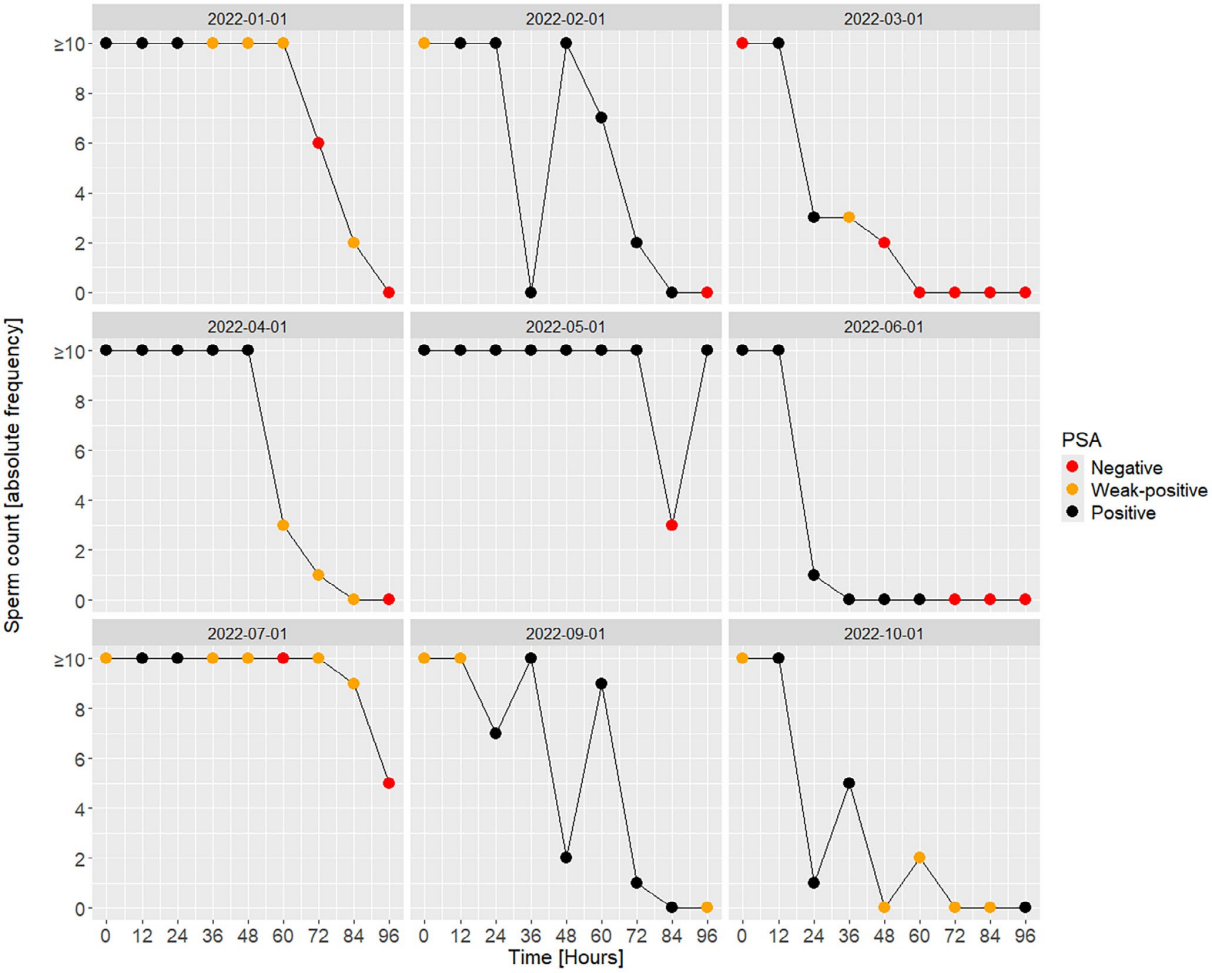
DE workflow—microscopy and PSA results. Line plot illustrating sperm cell counts of nine subject couples within study cohort over a period of 96 h with respective prostate‐specific antigen (PSA) test results (red = negative, orange = weak positive, black = positive). Couple 2022‐08‐01 is not available (i.e., PSA positive for entire study duration but no microscopy).

In three samples, positive PSA tests could still be found after 96 h (Figure 1). Test results in the baseline samples, taken before sexual intercourse, yielded inconsistent results, with 5 out of 10 positive tests (Figure S1). Three participants who tested positive after 96 h had already shown positive baseline samples. Sperm microscopy showed high variability within and between couples. In 2022‐08‐01, microscopy could not be performed due to incorrect sample processing. In 5 out of 9 couples, Figure 1 illustrates a decrease in sperm count over time (2022‐01‐01, 2022‐03‐01, 2022‐04‐01, 2022‐06‐01, and 2022‐07‐01); however, in the sample 2022‐05‐01, no decrease was observed except for one drop at T84. In 2022‐02‐02, a drop was observed at T36 with undetectable sperm counts, followed by declining sperm counts over time. In contrast, an alternating increase and decrease in sperm counts were noticed in couples 2022‐09‐01 and 2022‐10‐01. In three couples (2022‐03‐01, 2022‐06‐01, and 2022‐10‐01), fewer than 10 spermatozoa were detected after 12 h, while sperm cells were still detected at T96 in two couples. Microscopy of baseline samples was consistently negative.

A comparative analysis of PSA and microscopic investigation demonstrates that the respective results do not consistently align (Figure 1). A heat map summarizing the findings of PSA testing for all donors can be seen in the Data S1. For the couple 2022‐08‐01, all PSA tests were (weak) positive without deviation up to 96 h (Figure S3).

#### 3.1.2 Differential extraction—DNA yield estimation by real‐time PCR

**FIGURE 2:**
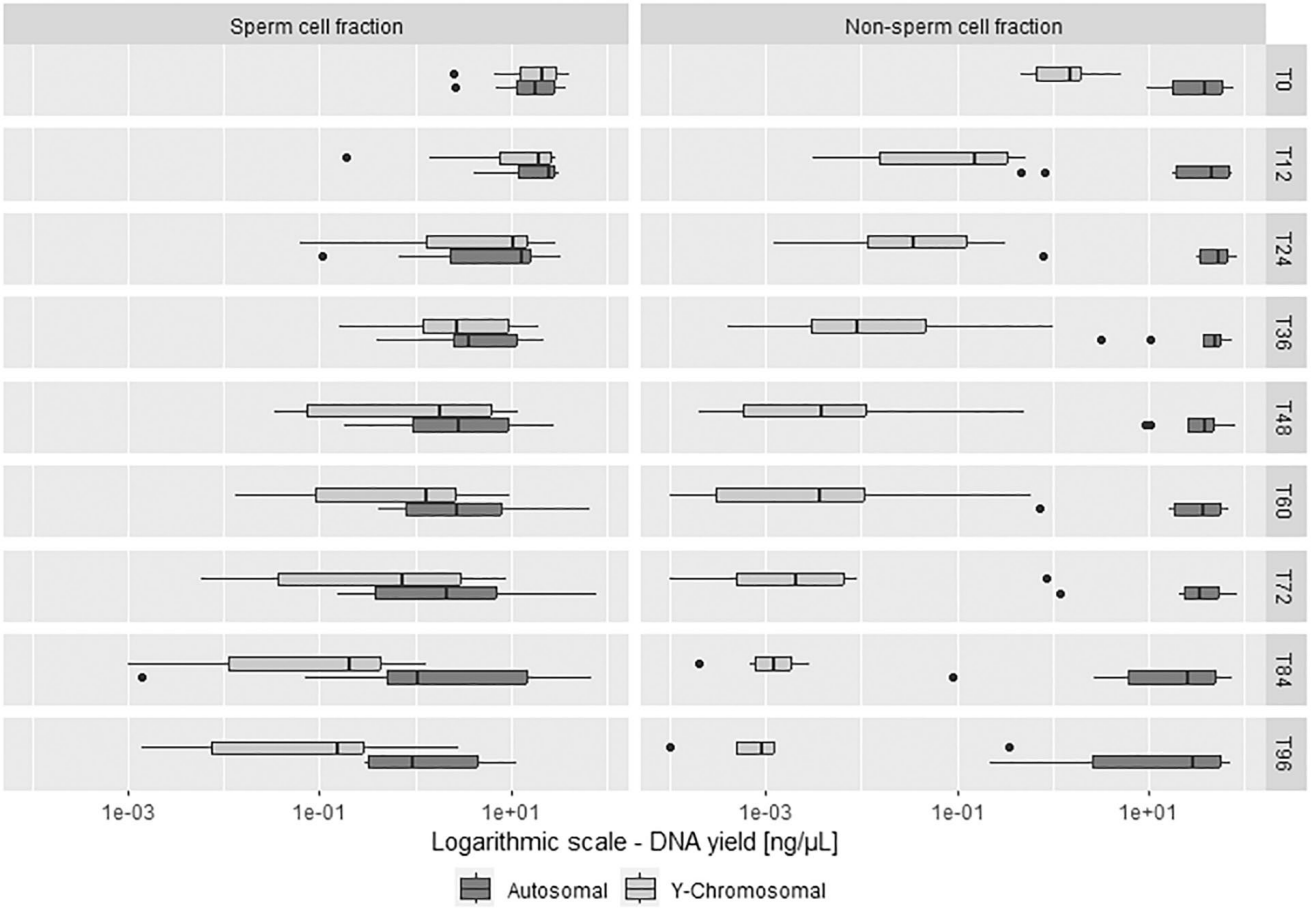
DE workflow—DNA yield estimation by real‐time PCR. Boxplots depicting autosomal and Y chromosomal DNA yield of sperm cell and epithelial fractions at different time points for all subjects.

DNA quantification was performed in triplicate for SF and NF (each *n* = 90). As expected, the quantities of autosomal and Y‐chromosomal DNA of the SF diminished from T0 to T96. During the initial 36 h, the autosomal and Y‐chromosomal DNA quantities of the SF are observed to maintain a balanced 1:1 ratio, and hence, allow for a clear distinction between the SF and NF (Figure 2). For the NF, a substantial reduction was only observed in Y‐chromosomal DNA yield (*p* ≤ 0.05, Figure 2).

The linear regression model revealed a statistically significant relationship between TSI and couple on Y‐chromosomal DNA yield in the SF (*p* ≤ 0.05). An average increase of 12 h correlated with a reduction in male DNA quantity (SF) by approximately 50% (*p* < 0.001). The regression results revealed no significant effect of showering on the relative change in DNA yields across two time points (*p* = 0.452). The total number of showers and their respective TSI per female study participant are listed in Table S3. In addition, no significant differences in relative decrease rates among the various time point intervals were observed through pairwise post‐hoc contrast analysis of estimated marginal means of male DNA, indicating that the relative decrease in DNA quantity remains constant over time.

#### 3.1.3 Differential extraction—Genotyping

Complete single male DNA profiles were generated for samples taken directly after sexual contact (T0), except for 2022‐07‐01 (mixed profile with major contribution). From T12 to T96, there were female‐only (6%) and male‐only (13%) profiles as well as mixed profiles (81%) with either male (38%) or female (37%) major components or balanced contribution (25%) in the SF. 82% were complete, and 6% were comparable, resulting in 12% uninterpretable or no male profiles (Figure 6). Even at 96 h, 7 out of 10 couples had still interpretable male profiles, exceeding the sampling time window of 72 h for sexual assault victims (Figure 6).

In the case of the female‐only profiles of the SF samples, no male contribution could be manifested, despite sperm having previously been detected by microscopy in four samples (i.e., T12 and T84 in 2022‐01‐01, T84 in 2022‐05‐01, and T84 in 2022‐07‐01). No male profile could be generated in sample T96 from 2022‐03‐01, and no sperm was detected microscopically (Figure 6). In contrast, male profiles were generated more frequently when no sperm was detected by microscopy. Despite the visual absence of sperm, 16 complete and two comparable male profiles were obtainable. In comparison, three male profiles were uninterpretable (Figure 6). The different sample fractions taken for DE and microscopy, respectively, will contain varying proportions of male cellular material.

Admixtures of female alleles observed within SF profiles were likely due to incomplete fraction separation during DE. Except for a few minor admixtures, male DNA was not visible in autosomal STR profiles of the epithelial fractions. These profiles comprised the unquestioned female reference profiles (data not shown).

#### 3.1.4 Laser microdissection—Image analysis and genotyping

**FIGURE 3:**
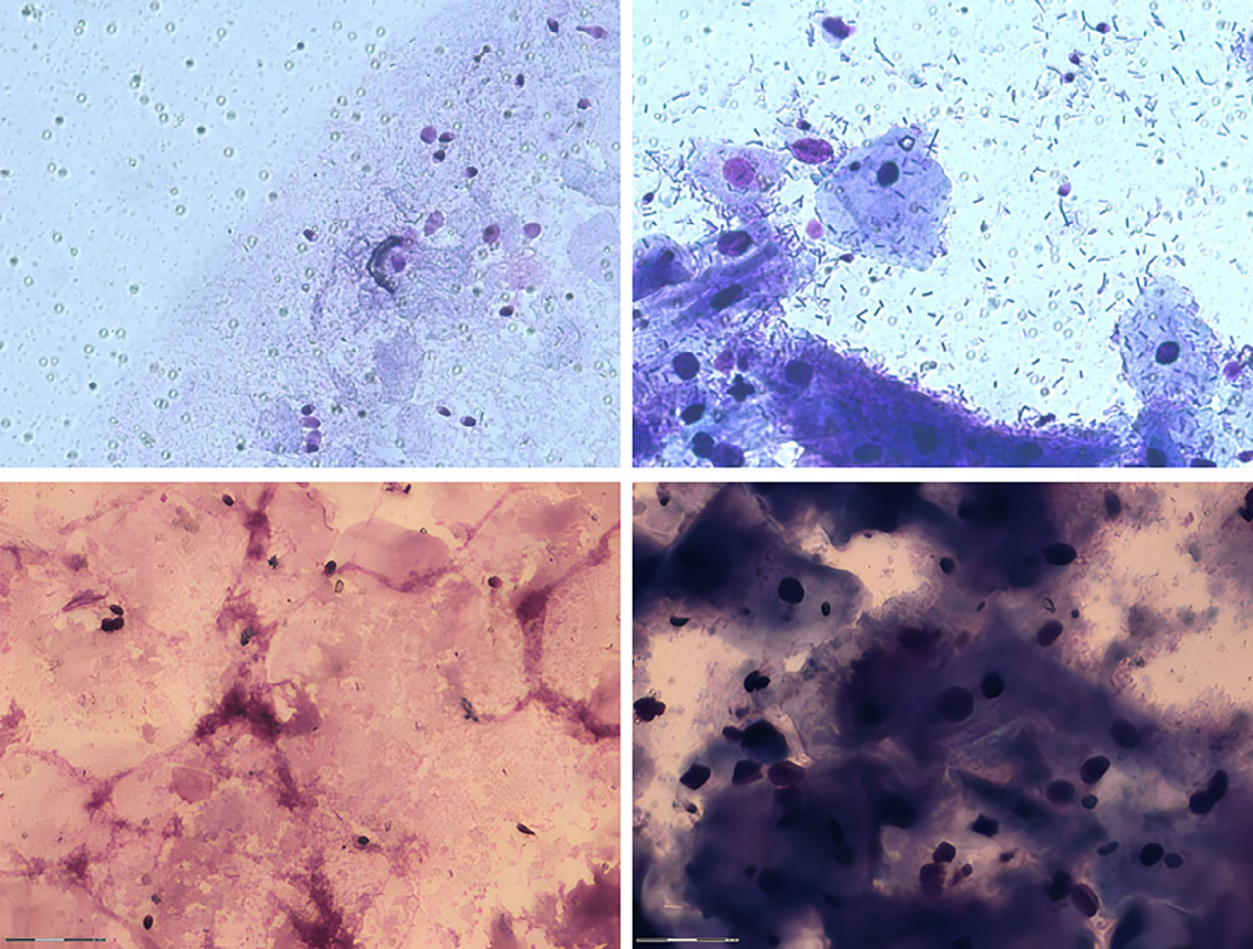
LCM workflow*—*microscopic image gallery. HE‐stained slide containing sperm cells with (left) and without (right) prior proteinase K digestion in 2022‐06‐01 (above) and 2022‐10‐01 (below).

Similar to microscopic examination, the slides lacking the prior mild digestion exhibited an abundance of epithelial cells, hence impeding the recovery of sperm. The lack of sufficient sperm counts for genotyping and the disproportionate time required per slide emphasize the need for enrichment strategies. Fortunately, the slides containing the enriched spermatozoa exhibited preserved sperm cells with no apparently altered morphology and were not compromised by an enhanced background due to present cell debris and cellular scrap, except for 2022‐10‐01. The provided sample material from the latter couple exhibited a high degree of secretion and thus viscosity, accompanied by an abundance of surplus epithelial cells, which posed challenges for microscopic analysis, even after undergoing mild digestion (Figure 3).

A minimum of 50 spermatozoa were observed until 72 h among samples, except for two couples, where 50 sperm cells could be detected until 12 h (2022‐01‐01) and 36 h (2022‐03‐01). In 4/10 of the couples, no sperm was found at the 84‐h mark, and in 7/10 couples at the 96‐h mark; thus, no genotyping was conducted in 12% of samples. Genotyping results revealed male single‐source profiles (24%), female‐only profiles (5%), but also mixtures (62%; 20% female and 49% male major component and 31% balanced genetic contribution), and no profiles (9%). Of the profiles generated, 46% of male profiles were complete, 29% were comparable, while 13% were uninterpretable. Although sperm were counted in 10 samples (T12‐T96), no male profiles could be generated. In contrast, in 11 samples (at T84 and T96), no sperm were found and, as mentioned above, no genotyping was performed (Figure 6).

In contrast to the DE, fewer couples (3/10) had complete male profiles visible at 96 h, with some exceptions between, yielding uninterpretable profiles (i.e., T24 in 2022‐08‐01 and T24 in 2022‐09‐01). For 2022‐02‐01, interpretable male profiles could be obtained up to T84 (exceptions at T0, T24, and T36). For 2022‐03‐01, 2022‐04‐01, and 2022‐06‐01, the cut‐off to generate interpretable profiles was T72, except for T48 and T60 in 2022‐03‐01. For 2022‐07‐01, interpretable male profiles were obtained until T60, with a non‐interpretable male profile at T72. On the other side, samples from 2022‐01‐01 showed earlier cut‐offs with interpretable profiles up to T36 (Figure 6). As demonstrated for DE, the sampling window of sexual assault victims was exceeded for three couples, genotyping successfully the profile of interest.

#### 3.1.5 DEPArray™—Image analysis and genotyping

**FIGURE 4:**
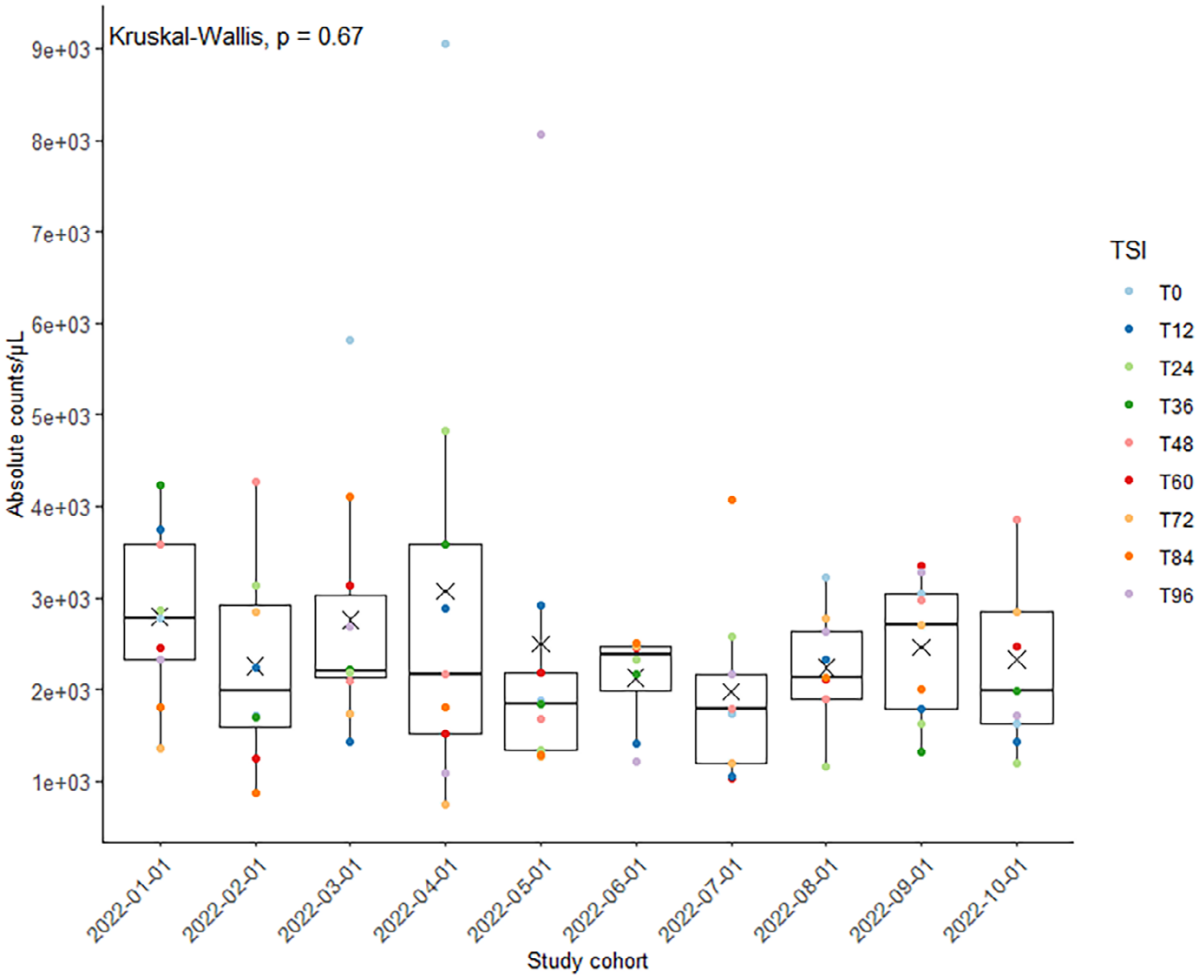
DEPArray™ workflow*—*released cellular material from swab heads. Box and scatter plot depicting total cell counts (absolute counts/μL) counted using Countess3 (*n* = 88 for 10 participants (study cohort) and 9 sampling time points (TSI)). For 2022‐02‐01 T96 and 2022‐07‐01 T0, no cell count was conducted, as no large cell pellet was visible. Kruskal–Wallis test revealed no statistical significance (*p* > 0.05).

The average number of cells counted from the swab heads was 2459 ± 1330 cells per μL (Figure 4). The statistical analyses did not yield any statistically significant findings concerning the absolute cell counts for the study cohort and TSI (*p* = 0.67 and 0.88, respectively), indicating continuous sampling amounts regardless of time and participants. Slight variations among participants and time points were considered due to the differing amount of adhering sperm and stochastic sampling variation.

**FIGURE 5:**
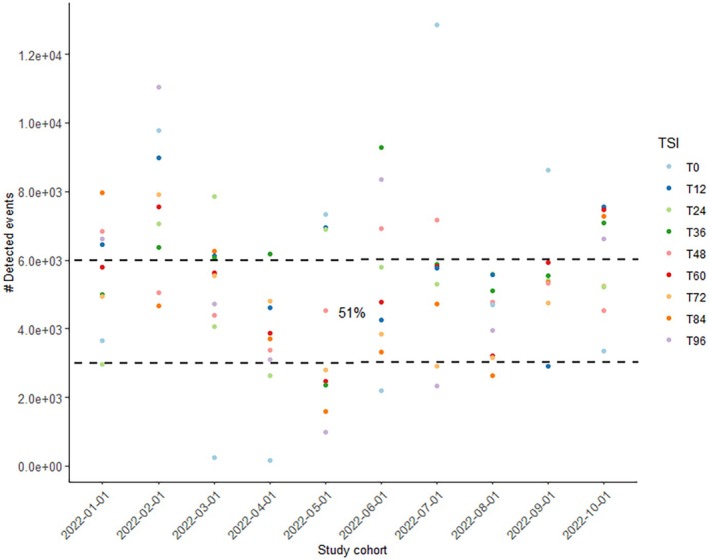
DEPArray™ workflow*—*detected events. Scatter plot illustrating detected events by CellBrowser™ software after DEPArray™ chip scan. Besides cells, events can include cell‐free nuclei or false‐positive signals. Dotted lines mark the anticipated cell load range from 3000 to the recommended maximum number of 6000 cells that should be analyzed per run. These criteria were met in 51% of the runs (*n* = 90 for 10 participants (study cohort) and 9 sampling time points (TSI)).

Due to technical limitations of the DEPArray™ (i.e., maximum cartridge capacity), 10,000 cells were stained with a maximum of 6000 cells loaded on the cartridge. Of all experiments performed (*n* = 90), 51% of the events detected by the CellBrowser™ software after the chip scan were within the anticipated loading range of 3000–6000 cells per cartridge [29]. In comparison, more than 6000 DAPI‐positive signals were detected in 33% and less than 3000 in 16% of the runs (Figure 5). Not considered are cell clusters that were unintentionally captured in the same “cage.” Fluctuations in cell count determination may arise due to user‐related factors, such as inadequate homogenization of the cell suspension and/or variability in pipetting small amounts. Furthermore, it is worth noting that post‐coital samples may exhibit variations in viscosity because of the presence of vaginal and seminal secretory components. T0 samples were classified as difficult to count, presumably because of the vast sperm count and amount of secretion (Figure 5).

**FIGURE 6:**
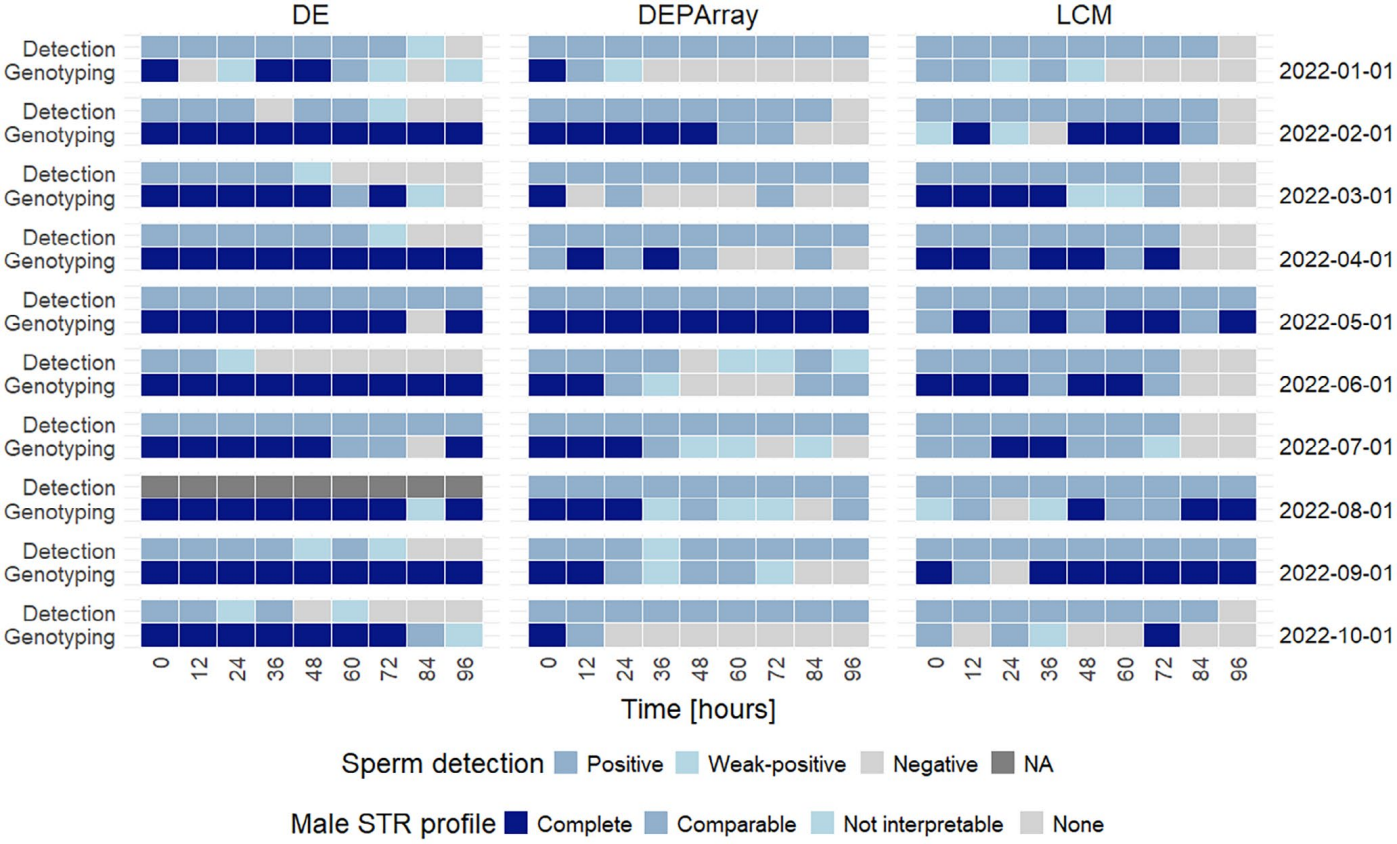
All methods*—*summarized sperm detection and genotyping outcome. Heat map comparing the sperm detection limits of the different methods with negative (no sperm), weak positive (1–2 sperm cells) and positive (≥3 sperm cells) as well as their respective genotyping outcomes. Solely male contribution was evaluated. Profiles were generally classified as single‐source or profiles obtained from mixed sources with male major or minor components. The latter was further categorized as “(nearly) complete,” “comparable,” and “not interpretable.” In 2022‐08‐01, microscopy could not be performed due to incorrect sample processing (NA, marked in gray).

In total, sperm cells could be effortlessly detected in the routability fraction and recovered up to 96 h in 9/10 couples, with no sperm found in 2022‐02‐01 T96 (Figure 6). In earlier time points, no sperm were detected only once (i.e., 2022‐06‐01 T48). Approximately 46 ± 10% of all detected events were routable with a minimum, median, and maximum value of 21%, 47%, and 71%, respectively. Of these, approximately 17 ± 6% were subsequently declared as non‐routable or went lost during routing owing to encountered (undetected) obstacles (i.e., cell cluster and particles), long distance‐to‐move (routing paths), and/or poor cell condition (not detectable). There was a weak to moderate positive (linear) correlation between total counts and non‐routability (Pearson 0.43, Spearman's rank 0.39), indicating that a rise in events might increase the proportion of non‐routable cells.

Interpretable male DNA profiles were generated for samples taken directly after sexual contact (T0) up to T12, derived from single‐source or mixtures with the male contributor as the major component. There were male (13%) but also female‐only (24%) profiles as well as mixed profiles (56%) with either male or female major components or balanced genetic contribution. Of these, male contribution was to 33% complete, 22% comparable, while 45% resulted in uninterpretable or no male profiles (Figure 6). In total, two DEPArray™ analyses showed no sperm; thus, no genotyping was performed (2022‐02‐01T96 and 2022‐06‐01T48). In 1/3 (*n* = 30) of the samples, sperm were detected but did not provide a male profile.

Nevertheless, until 96 h, in 3 out of 10 couples (i.e., 2022‐05‐01, 2022‐06‐01, and 2022‐08‐01), interpretable profiles could be generated (i.e., 100% complete and 69% and 39% comparable). Like the LCM and DE, the detection limits for male interpretable profiles obtained differed between couples and time points. For 2022‐04‐01, the cut‐off could be assigned to T84 (except for two samples at T60 and T72). In two couples (2022‐02‐01 and 2022‐03‐01), interpretable profiles could be obtained up to T72, again with exceptions (T12, T36, T48, and T60 for 2022‐03‐01). For 2022‐09‐01, the detection limit can be assigned to T60, except for T36. For 2022‐07‐01, the detection limit for interpretable male profiles was T36. For 2022‐01‐01 and 2022‐10‐01, a minimum detection limit of T12 was determined.

Figure 6 depicts a heat map summarizing the sperm detection and genotyping ability for each method. Here, it is illustrated that sperm detection in cell capture strategies is not accompanied by genotyping success due to limited quantities and that no sperm detection before DE does not mean that no male profile can be generated, even if it cannot be assigned to a specific cell type.

## 4 DISCUSSION

### 4.1 Sperm detection and persistence

In sexual assault studies, post‐coital sperm persistence is discussed controversially within the literature, with reproductive biology studies reporting 3–7 days in adults and 5 days in a child's vaginal vault [8, 48]. Consensual intercourse results suggest a maximum persistence of 9 days in the vagina, 12 days in the cervix, and 10 days using cervicovaginal smears [8]. This contrasts with retrospective studies on forensic assaults, indicating a sperm persistence of approximately 24–48 h [49, 50, 51]. We assume that differences in testing methodologies and evaluation criteria (e.g., microscopic or genetic examination), but also experimental settings (e.g., storage, sample material, and study design), case circumstances (e.g., nature of penetration or the presence of ejaculation), as well as individual variations (e.g., hygiene practice), might contribute to poor sperm persistence and discrepancies in study outcomes. In our case, differences in the methodology chosen might also explain the variation in the sperm detection and genotyping results reported. Irrespective, we detected spermatozoa up to 96 h, showing that both TSI and individuals significantly affected the quantity of male DNA. Variations among donors are likely attributable to variations in initial sperm quantity and quality (depending, e.g., on age, lifestyle, and medication), variances in the female reproductive tract and contraceptive method used, variations in hygiene practices (e.g., frequency and duration of showering/bathing), and changes in activity levels (e.g., visits to swimming/thermal pools, occupational/leisure activities). In this study, only showering frequency was addressed, which did not significantly affect the male DNA yield from intravaginal swabs. Nevertheless, it is plausible to hypothesize that donor dependency could still be influenced by the parameters mentioned above, and by post‐coital hygiene behavior. Ensuring total standardization via tightly controlled study conditions and a broader range of variables in the survey (e.g., toilet usage and physical activities) would impose significant restrictions on the participants' daily routines, likely leading to less participation and underrepresenting reality.

### 4.2 Suitability of self‐sampling

For self‐sampling, it is essential to note that the study participants did not possess medical qualifications, and their adherence to and compliance with study protocols, including the sequence and time of swabbing, was presumed but could not be checked (except for adherence to abstinence by the baseline sample). Incorrect self‐reporting can lead to erroneous results due to multiple sources of bias already reported elsewhere [52]. For medical examiners, Astrup et al. [48] described the optimal intravaginal sampling sites of spermatozoa in healthy individuals after consensual sexual intercourse. In contrast, Bahamondes et al. [53] summarized that self‐sampling of vaginal fluid was equivalent to sampling done by a nurse in obtaining information on semen exposure measured by PSA. While the authors claimed some limitations in their study design, the results at least indicated that both sampling methods are highly sensitive. In our study, however, some isolated DNA yields, cell counts, and genotyping results might indicate irregular (i.e., sampling performed differently at each time point) or potentially erroneous sampling (i.e., swab order and time point mixed up). However, these irregularities may also be the result of stochastic effects. Counts from cellular material released from the swab heads did not reveal any significant difference among couples and sampling time points, indicating that self‐sampling and randomization of swabs was, in principle, an appropriate approach. However, it is important to acknowledge that cell‐counting methodologies, whether manual or automated, remain estimations, which are additionally influenced by factors such as pipetting inaccuracies, presence of cell aggregates or debris, and different cell type characteristics (e.g., ≤4 μm), as evidenced by the discrepancies between the input cell counts and the detected events in the DEPArray™ analysis. The latter defines positive events, inter alia, based on the detection channel chosen (e.g., DAPI) and the threshold settings applied.

### 4.3 Standard workflow—PSA Testing and DE

From the post‐coital samples, we observed 100% positive PSA test results until T36, 90% until T48, and 80% until T60. So, positive PSA results were obtained long after sexual intercourse, except for the T48 PSA result of one female participant (2022‐03‐01), who visited a thermal bath after the first 24 h. It is important to note that post‐coital samples after consensual sexual intercourse with intravaginal ejaculation were used instead of casework or mock samples, and that no actual PSA concentrations were determined in this study. In the literature, the limit of PSA detection is reported to be highly variable depending on the study setup (i.e., prospective after unprotected intercourse or inoculation of the vagina with semen vs. retrospective in casework) and the type and sensitivity of the PSA test used. While PSA was generally determined to be detectable up to several days after semen exposure, studies also described that positive test results considerably decreased within the first 10–48 h [9, 53, 54, 55, 56, 57].

In one instance, a false‐negative PSA test was observed in a T0 sample, which the high‐dose hook effect can explain [58], as microscopic and sperm genotyping results were positive. Unexpectedly, we found 50% false‐positive PSA results in the baseline samples taken before sexual intercourse, whereas microscopy, quantification, and genotyping did not reveal any sperm or male DNA. Given that three of the women who tested positive in the baseline sample also tested positive after 96 h following sexual intercourse, it is plausible that this could be attributed to naturally existing (background) PSA levels, such as those found in vaginal fluid, rather than the presence of seminal fluid. Thus, evaluating PSA test results poses a further challenge, as they can be positive even without seminal fluid. Although it is known that PSA is present in lower concentrations in other bodily fluids [59], such as vaginal fluid [60, 61], existing studies indicate that concentrations of endogenous PSA identified in a vaginal fluid are too low to be misclassified as resulting from semen exposure [57, 62]. An exception was described by Denison et al. [63], who found a single positive result correlated with the menstrual cycle (i.e., 3 days before or during menstruation). In contrast, Kafarowski et al. [64] found no positive PSA results in vaginal swabs taken during the entire menstrual cycle. Based on the provided participant information, the menstrual cycle could be excluded as a factor contributing to the observed results. However, at least the relevance of false positive acid phosphatase reactions from vaginal samples has been described elsewhere [65].

DE‐processed samples provided a profile for each sample analyzed, with the highest proportion of interpretable male profiles. Compared to single‐cell technologies, the DE approach allows the assessment of the entire sample material (including cell‐free DNA and male non‐sperm cells), although there is still a presumable loss of material due to the extraction process itself. However, the SF and NF fractions can be impacted by carryover of male and female cells, while in this study, more epithelial cell carryover was found in the later TSI time points, possibly due to sperm depletion. Direct comparison of genotyping results with the ability to detect sperm showed that male interpretable profiles were more often generated, but no sperm were identified than vice versa, likely due to the different sample fractions used for either presumptive or confirmatory tests.

### 4.4 Impact of post‐coital sample material on cell detection and genotyping in capturing workflows

For the cell capture workflows, genotyping yielded interpretable male profiles in 52% (DEPArray™) and 65% (LCM), with considerable variation between time points and individuals, likely due to different sub‐sample volumes processed and technical limitations later addressed, where individual components (e.g., initial sperm counts) might have a greater impact. Anslinger et al. [66] reported that the success of cell recovery with the DEPArray™ is dependent on the quality of the cells, while the genotyping is dependent on DNA quantity and quality. Since the generation of single‐cell profiles from intact cells is already challenging due to the small template quantities, it is made more difficult by poor DNA quality. Thus, a deterioration in quality can lead to higher dropout rates or entirely negative results. This can be partially offset by the number of available, successfully isolated target cells, as Vernarecci et al. [67] demonstrated.

Regarding quality, a comparative analysis of post‐coital samples processed with LCM and DE by Elliott et al. [68] suggested that the TSI played a substantial role in determining the success rates of sperm genotyping. The increased duration of TSI was presumed to lead to a higher occurrence of apoptosis, which in turn should have caused DNA degradation. Even if the sperm have undergone nuclear cleavage but not the subsequent stages of apoptosis (i.e., destruction of sperm heads), they maintained their morphologically intact appearance [68]. Further, the increased exposure to the vaginal fluid [68] and corresponding microflora in the woman's body [69] and contraceptive methods with barriers or hormones [70, 71] have also been found to impair sperm quality, while Astrup et al. [48] could not find any correlation. While this would apply to all procedures within this study, storage time due to delayed processing affecting cell quality and the technical limitations in the number of cell assessments affecting cell quantity may have favored degradation and, thus, lower genotyping effects in the cell capturing techniques. In addition, Di Martino et al. [72] stated that intact‐looking sperm heads from smears processed by laser microdissection might harbor degraded DNA due to the method‐specific fixation and staining procedures.

That prolonged exposure to vaginal fluid can hamper sperm genotyping, is further proposed by intricate interactions between sperm and exogenous female DNA from lysed cells that can adhere to the sperm head. This can lead to the activation of nucleases inside the spermatozoa, causing the fragmentation of sperm cell DNA and ultimately leading to apoptosis [73, 74]. Consequently, the presence of relatively high numbers of sperm cells does not assure good‐quality DNA profiles [68], and the impact of storage could be considered. While female DNA can (mostly) be removed from the sperm during DE, the single extraction step used in LCM or DEPArray™ techniques requires intact cells and, therefore, cannot selectively remove female DNA attached to the sperm [68, 74]. In general, the analysis of cell‐free DNA is not addressed when utilizing capture technologies, except perhaps for adherent DNA that has not been washed away, likely to explain differences in genotyping outcomes among methods (e.g., presence of mixtures and profile completeness).

### 4.5 Cell detection and capturing workflow—Laser microdissection

Without prior sperm cell enrichment, application of the LCM was challenging or even impossible because (1) spermatozoa were undetectable due to the mass of epithelial cells or (2) too few sperm cells were available for profiling due to the small amount applied to the slides. Increasing the volume or the application area would have substantially impacted the time required per slide. Only after enrichment did the LCM method yield appropriate sperm counts to generate solely complete or incomplete male profiles from single or mixed sources, while occasionally, no profiles were generated from recovered cells. At this point, it should be noted that the effect of proteinase K digestion on the premature lysis of spermatozoa is conversely discussed in the literature [75]. However, Tereba et al. [76], Greenspoon et al. [77], and Hennekens et al. [75] showed minimal carryover from sperm cell DNA into the NF and no indication of simultaneous sperm and epithelial cell lysis in the absence of DTT with proteinase K concentrations below 300 μg/mL when applying DE. Thus, the implementation of sperm cell enrichment procedures by, for example, mild lysis, has the potential to enhance the efficacy of and ideally minimize the duration required for spermatozoa detection and retrieval.

In this study, a maximum of 50 cells were sectioned, with the caveat that this number was contingent upon availability. This number was chosen as pre‐tests using fresh ejaculate yielded interpretable profiles with 50 cells, while microscopy with 90 slides was considered a time strain, as also noticed in other studies [28, 40]. Although the literature reports considerable variation in the number of sperm cells needed to achieve complete profiles, ranging from 30 to 150 sperm cells [24, 26, 40], 50 cells were generally sufficient to obtain complete STR profiles in this study. Discrepancies between studies can be explained by the characteristics of the sample material (such as age and quantity), aspects related to fixation or staining, the specific device employed (including the direction of cutting with or against gravity, as well as the type of laser used), and the method of extraction and amplification. The presence of occasional admixtures is likely attributed to the co‐isolation of surrounding epithelial or blood cells. When a sample has a substantial quantity of diverse, intermixed, or faulty stained cell types, the process of detecting and isolating the desired cells can be laborious, time‐intensive, and yield mixed profiles. Moreover, when cells are nearby, it may be advisable to eliminate the non‐target cells before collecting the target cells. This can be achieved by cutting around the target cells, yielding a clear‐cut gap between desired and undesired cellular material, or selectively ablating undesired cells with precise laser shots [28]. In addition, potential cell‐free female DNA could be a reason for the occurrence of mixed profiles, which would be accepted if sufficient sperm cells were available. No profiles were considered the result of handling failures or technical issues (e.g., cell loss, compromised DNA quality by laser application, and non‐lysed spermatozoa) or simply due to stochastic events commonly encountered with LT‐DNA samples. In addition, it was observed that there was a lack of (enough) detectable sperm cells in some slides, particularly during the later time intervals (T84 and T96).

Besides being a substantial time investment, it might be desirable to disperse more sample material and to isolate as many cells as possible for post‐coital or casework samples with higher TSI. For this, using automated methods and/or deep learning strategies with comprehensively trained convolutional neural networks effectively increases the speed of identifying target cells while mitigating the potential sampling bias that may arise from the individual users' subjective cell selection [40, 78, 79].

### 4.6 Cell detection and capturing workflow—DEPArray™

A key advantage of DEPArray™ technology is that it automates image acquisition and target cell detection. However, it is important to consider the technical limitation of the cartridge capacity of the DEPArray™ technology compared to manual microscopy, which allows for the analysis of only 3000–6000 cells at once and requires prior cell counting. Thus, not only about ¼ of the total sample was examined, which probably represents an underestimated number of sperm cells present in the initial sample, but may also lead to too few or too many loaded cells due to discrepancies in cell count determination. This underscores the necessity of pre‐enrichment of sperm cells to eliminate superfluous epithelial cells, as employed in LCM analysis. However, the utilization of proteinase K digestion is likely unsuitable for DEPArray™‐mediated recovery due to the requirement of preserved cell membrane integrity with intact protein targets for staining and routing, which needs to be proven. Moreover, excess cellular debris or fragments may affect the cells' navigability within the cartridge. As introduced by Williamson et al. [30], it can be confirmed that using a filtering procedure can avoid epithelial cell saturation, but it can be a labor‐intensive process that carries the potential risk of losing desired sperm likely adhering to epithelial cells and does not always yield the anticipated positive outcome [29]. Nevertheless, DEPArray™ technology was the only method that allowed us to detect sperm in 9/10 of the couples at T96, even without a prior sperm enrichment strategy. Compared to LCM, only two DEPArray™ runs had no sperm cells, hence no genotyping was performed.

The utilization of the DEPArray™ resulted in male profiles from a single source up to T12, while after T24, mainly female‐only profiles or mixed profiles of different compositions were generated. No profiles were generated in 8% of samples, revealing cell loss or less efficient DNA extraction and subsequent stochastic effects when analyzing small amounts of sperm cells [19]. Williamson et al. [30] identified and recovered sperm cells at each post‐coital interval from 12 to 96 h, with male profiles being the only ones until 24 h or the main component until 72 h. At the 96‐h, the group could uniquely identify one sperm and recovered 12 cells, resulting in a mixed profile with minor male donor contribution. While the detection rate is consistent with our results, their genotyping results contrast ours, which often revealed no interpretable profiles after 72 h. This suggests that endeavors to enrich sperm are crucial, even if they are not yet fully developed. Until then, for casework applications, it is advisable to perform at least duplicate runs or prior sample filtering for DEPArray™ analysis, particularly when there is a substantial quantity of vaginal epithelial samples. Nevertheless, it should be emphasized that we obtained interpretable profiles in 3/10 couples up to 96 h without any enrichment strategy.

However, what can be expected from the profile quality of DEPArray™‐derived cells is well‐studied, and the underperformance in genotyping outcomes, especially at later TSI, was first surprising, as Schulte et al. [19, 29] revealed that single cells could already yield approximately 25–30% profile completeness with regard to the diploid reference profile. In contrast, pools of 5, 10, and 20 cells resulted in 50%, 80%, and 90% profile completeness, respectively [29]. One has to keep in mind that in comparison to an autosomal cell, in theory, two sperm cells would be required to span the entire genetic information cumulatively, but only if the haploid cells differ in their genetic composition. Further, mock sample preparations were found suitable for sperm detection, separation, and genotyping with almost complete profiles up to a 1:1000 sperm‐to‐epithelial cell ratio with comparable recoveries, which was also reported in other studies [18, 30]. Therefore, sufficient sperm counts should have been collected from post‐coital samples to obtain at least mixed profiles with incomplete male profiles in the minor component. Presumably, however, mock samples cannot reflect the actual composition of the post‐coital samples, which should be addressed in further studies. Elliott et al. [69] already reported a difference in processing post‐coital samples versus artificial mixtures, especially in receiving mixed profiles. So far, most single‐cell groups have used fresh (mock) artificially mixed samples and mainly processed immediately [18, 30]. In addition, technical and stochastic issues such as cell loss during volume reduction should be considered, while cells were verified as parked prior to recovery.

Mixed profiles could be explained by sperm cells collating with epithelial cells, epithelial‐cell debris, or cell‐free DNA. If two cells form a cluster and are not microfluidically separated, both fall into the nearest electric field minimum and, thus, into one cage. However, these clusters were still recovered since the improved epithelial‐to‐sperm ratio of only two cells should yield a mixed but evaluable profile. The isolation of cell groups (comprising single and clustered sperm cells) was generally implemented to enhance the likelihood of successful genotyping, since these also would be isolated in forensic casework. Given the data provided for DE, the minor contributor remained identifiable up to a sperm‐to‐epithelium ratio of 1:10 [11]. As a result, the focus was on recovering as many sperm cells as possible while potentially collecting adhering epithelial cells. Concerning false‐positive signals, we observed that the software recommended more signals as spermatozoa, especially at later post‐coital intervals (T72‐T96), with single nuclei, antibody aggregates, obstacles, or small blood‐cells declared APC‐positive. Events that were identified as possible sperm were collected to obtain the maximum amount of genetic information, considering the low number of sperm expected at later TSI (≥72 h), as also described by Williamson et al. [30]. The occurrence of “no male profiles” can thus be explained by the limited number of spermatozoa at these time points and by unintentionally recovering false‐positive signals.

Although naturally occurring loss of biological material applies to all methods, DEPArray™ is a method where cell loss can be tracked. Regarding routability, approximately 50% of the detected events were deemed non‐routable, hence rendering them unavailable for investigation, even though cells of interest would have been available. After screening routablecells, the selected ones are directed toward the designated parking chamber and recovery exit. However, potential risks are associated with the “moving” process, including the possibility of cells becoming lost or trapped due to impediments that were not detected, such as cell clusters or particles. Additionally, the length of the routing paths and the cells' condition can contribute to potential issues during movement [19]. In the present investigation, spermatozoa were occasionally displaced during the parking phase, with at least one loss during the subsequent recovery process, as they transitioned from the parking chamber to the recovery exit. Moreover, suppose an instrument failure occurs at a specific stage after the sample has been loaded (which fortunately did not occur during the study), in this case, the biological material becomes irretrievable within the disposable cartridge [30]. The statement above holds for cells that are non‐routable or have become lost. Thus, the user has a keen interest in the manufacturer overcoming the instrumental limitation in future versions.

Table S4 summarizes the technical data of the three methods regarding sample type, time, and handling.

## 5 CONCLUSION

Conventional DE is an efficient and well‐established cell enrichment tool for separating (standard) sexual assault mixtures, enabling single‐source male/female or more easily interpreted mixture profiles while being simple, fast, and cost‐efficient. However, the utilization of a more demanding single‐cell separation technique, such as LCM or DEPArray™, can prove advantageous in (1) DE‐inappropriate scenarios involving same cell mixtures or multiple‐contributor crimes (including legitimates) or (2) supportive strategy when conventional approaches (i.e., PSA, microscopy, and DE) yield no, inconclusive, or insufficient outcomes. Since decisions for or against DE may be based on PSA and microscopic results (while at least both should be conducted), misguiding data can result in “standard” extraction and likely in loss of the minor contributor. Since half of the study's baseline PSA tests were false positives, more careful attention to their interpretation should be given. Thus, credible image analysis by LCM or by DEPArray™ coupled with specific cell sorting offers distinct benefits, as it enables (1) prior detection and identification of the (desired) cell type and (2) (ideally) subsequent assignment of profiles to contributors, independent of the biological mixture composition. Particularly in the case of disputed sexual assaults, the assignment of the male profile to a specific cell type, such as epithelial or sperm cells, can be decisive in court.

The successful implementation of cell capture technologies for sexual assaults also depends on the initial sperm counts, highlighting the need for sperm enrichment and purification strategies prior to sorting/subsampling when processing vaginal swabs. As implemented for the LCM, enabling sufficient sperm counts while saving time, we still see a demand for the DEPArray™ technology to mitigate the presence of excessive female cellular material with respect to restricted cartridge capacity. Combined with an adjusted sample preparation protocol, including improved sample homogenization and a second counting step, the ideal number of cells (that should not exceed the maximum of 6000 cells) can be processed per run, realizing its full potential and minimizing costs. Concerning time capability, the DEPArray™'s automatic image analysis was highly valued in comparison to sperm microscopy and LCM, which could be principally counteracted by deep learning strategies and further automation steps.

To bridge the DEPArray™ technology to sexual assault applications, it would be desirable to incorporate the optimizations mentioned above on fresh and stored artificial‐mixed mock and post‐coital samples, ensuring the availability of sufficient sperm counts and addressing storage effects in the context of DNA degradation. Lastly, it would be intriguing to compare further cell separation methodologies, such as micromanipulation or FACS, with the inclusion of their suitability for such specimens.

## FUNDING INFORMATION

This research was partly funded by the Research Funds of the University of Basel (No. 3MB1011).

## CONFLICT OF INTEREST STATEMENT

The authors declare no competing financial and non‐financial interests.

## ETHICS STATEMENT

This study involved samples from voluntary participants obtained in a monocentric prospective and descriptive study with risk category A that was approved by the local ethics committee (ID 2021‐02097). All procedures performed in studies involving human participants were in accordance with the ethical standards of the institutional and/or national research committee and with the 1964 Helsinki Declaration and its later amendments or comparable ethical standards.

## INFORMED CONSENT

Written informed consent was obtained from all participants.

## Supporting information


Data S1.

## Data Availability

The datasets generated during and/or analyzed during the current study are available from the corresponding author on reasonable request. The study results were presented at the 44th Spurenworkshop, March 7–9, 2024, in Frankfurt am Main, Germany; the SGRM Sommertagung, June 7–8, 2024, in Sion, Switzerland; and at the 30th International Congress of the International Society for Forensic Genetics; September 9–13, 2024, in Santiago de Compostela, Spain.

## References

[1] Inci F , Ozen MO , Saylan Y , Miansari M , Cimen D , Dhara R , et al. A novel on‐chip method for differential extraction of sperm in forensic cases. Adv Sci. 2018;5(9):1800121. 10.1002/advs.201800121 PMC6145299 30250782

[2] Nakagawa T , Doi M , Nishi K , Sugahara T . Advantages of filtration method for sperm‐DNA genotyping in sexual assault cases. Leg Med. 2022;54:101988. 10.1016/j.legalmed.2021.101988 34915337

[3] Gill P , Jeffreys AJ , Werrett DJ . Forensic application of DNA “fingerprints”. Nature. 1985;318:577–579. 10.1038/318577a0 3840867

[4] van den Berge M , Sijen T . Development of a combined differential DNA/RNA co‐extraction protocol and its application in forensic casework. Forensic Sci Int Rep. 2022;5:100261. 10.1016/j.fsir.2022.100261

[5] Clark C , Turiello R , Cotton R , Landers JP . Analytical approaches to differential extraction for sexual assault evidence. Anal Chim Acta. 2021;1141:230–245. 10.1016/j.aca.2020.07.059 33248657

[6] World Health Organization . Guidelines for medico‐legal care for victims of sexual violence. Geneva: World Health Organization; 2003.

[7] O'Donohue WT , Schewe PA , editors. Handbook of sexual assault and sexual assault prevention. London, UK: Springer; 2019. 10.1007/978-3-030-23645-8

[8] Suttipasit P . Forensic spermatozoa detection. Am J Forensic Med Pathol. 2019;40:304–311. 10.1097/PAF.0000000000000517 31687979

[9] Egger S , Vöhringer C , Währer J , Schulz I . Technical note: comparison of forensic swabs for intravaginal sampling. Sci Justice. 2022;62:418–423. 10.1016/j.scijus.2022.05.006 35931447

[10] Horsman KM , Barker SLR , Ferrance JP , Forrest KA , Koen KA , Landers JP . Separation of sperm and epithelial cells in a microfabricated device: potential application to forensic analysis of sexual assault evidence. Anal Chem. 2005;77:742–749. 10.1021/ac0486239 15679339

[11] Vuichard S , Borer U , Bottinelli M , Cossu C , Malik N , Meier V , et al. Differential DNA extraction of challenging simulated sexual‐assault samples: a swiss collaborative study. Investigative Genet. 2011;2:11. 10.1186/2041-2223-2-11 PMC3119174 21542912

[12] Vignani R , Scali M , Liò P . Molecular markers and genomics for food and beverages characterization. In: Dash HR , Shrivastava P , Lorente JA , editors. Handbook of DNA profiling. Sinapore: Springer; 2022. p. 889–909. 10.1007/978-981-16-4318-7_43

[13] Timken MD , Klein SB , Kubala S , Scharnhorst G , Buoncristiani MR , Miller KWP . Automation of the standard DNA differential extraction on the Hamilton AutoLys STAR system: a proof‐of‐concept study. Forensic Sci Int Genet. 2019;40:96–104. 10.1016/j.fsigen.2019.02.011 30785062

[14] Woolf MS , Cunha LL , Hadley KC , Moffett RP , Landers JP . Towards an affinity‐free, centrifugal microfluidic system for rapid, automated forensic differential extraction. Anal Chim Acta. 2023;1249:340826. 10.1016/j.aca.2023.340826 36868762

[15] Goldstein MC , Cox JO , Seman LB , Cruz TD . Improved resolution of mixed STR profiles using a fully automated differential cell lysis/DNA extraction method. Forensic Sci Res. 2020;5:106–112. 10.1080/20961790.2019.1646479 32939426 PMC7476624

[16] Huffman K , Ballantyne J . Single cell genomics applications in forensic science: current state and future directions. iScience. 2023;26:107961. 10.1016/j.isci.2023.107961 37876804 PMC10590970

[17] Prajapati S , Rajmane P , Jayakrishna P , Nair MS , Kshirsagar P , Meshram M . Application and utility of alternative methods in isolation of pure cells from forensic biological mixtures in modern‐day: a review. J Forensic Sci Res. 2021;5:41–47. 10.29328/journal.jfsr.1001026

[18] Fontana F , Rapone C , Bregola G , Aversa R , de Meo A , Signorini G , et al. Isolation and genetic analysis of pure cells from forensic biological mixtures: the precision of a digital approach. Forensic Sci Int Genet. 2017;29:225–241. 10.1016/j.fsigen.2017.04.023 28511094

[19] Schulte J , Marciano MA , Scheurer E , Schulz I . A systematic approach to improve downstream single‐cell analysis for the DEPArray™ technology. J Forensic Sci. 2023;68(6):1875–1893. 10.1111/1556-4029.15344 37497755

[20] Watkins DRL , Myers D , Xavier HE , Marciano MA . Revisiting single cell analysis in forensic science. Sci Rep. 2021;11(1):7054. 10.1038/s41598-021-86271-6 33782417 PMC8007698

[21] Theunissen GMG , Gibb A , Lin PKT , Rolf B , Forat S , Jäger R . DNA profiling of single sperm cells after whole genome amplification. Forensic Sci Int Rep. 2021;4:100240. 10.1016/j.fsir.2021.100240

[22] Huffman K , Hanson E , Ballantyne J . Recovery of single source DNA profiles from mixtures by direct single cell subsampling and simplified micromanipulation. Sci Justice. 2021;61:13–25. 10.1016/j.scijus.2020.10.005 33357824

[23] Huffman K , Hanson E , Ballantyne J . Y‐STR mixture deconvolution by single‐cell analysis. J Forensic Sci. 2023;68(1):275–288. 10.1111/1556-4029.15150 36183153

[24] Vandewoestyne M , Deforce D . Laser capture microdissection in forensic research: a review. Int J Leg Med. 2010;124:513–521. 10.1007/s00414-010-0499-4 PMC2952761 20680318

[25] Vandewoestyne M , Goossens K , Burvenich C , Van Soom A , Peelman L , Deforce D . Laser capture microdissection: should an ultraviolet or infrared laser be used? Anal Biochem. 2013;439:88–98. 10.1016/j.ab.2013.04.023 23643622

[26] Kongruang A , Shotivaranon J , Areesirisuk P . DNA profiling of microdissected spermatozoa. Genomics and Genetics. 2021;14:9–17. 10.14456/gag.2021.2

[27] Han JP , Yang F , Xu C , Wei YL , Zhao XC , Hu L , et al. A new strategy for sperm isolation and STR typing from multi‐donor sperm mixtures. Forensic Sci Int Genet. 2014;13:239–246. 10.1016/j.fsigen.2014.08.012 25240154

[28] Vandewoestyne M , Deforce D . Laser capture microdissection for forensic DNA analysis. Forensic Sci Int Genet Suppl Ser. 2011;3:e117–e118. 10.1016/j.fsigss.2011.08.058

[29] Schulte J , Caliebe A , Marciano M , Neuschwander P , Seiberle I , Scheurer E , et al. DEPArray™ single‐cell technology: a validation study for forensic applications. Forensic Sci Int Genet. 2024;70:103026. 10.1016/j.fsigen.2024.103026 38412740

[30] Williamson VR , Laris TM , Romano R , Marciano MA . Enhanced DNA mixture deconvolution of sexual offense samples using the DEPArray™ system. Forensic Sci Int Genet. 2018;34:265–276. 10.1016/j.fsigen.2018.03.001 29602061

[31] Fedlex . Ordinance on human research with the exception of clinical trials (HRO). https://www.fedlex.admin.ch/eli/cc/2013/642/en. Accessed 5 Jun 2025.

[32] Fedlex . Human Research Act (HRA). https://www.fedlex.admin.ch/eli/cc/2013/617/en. Accessed 5 Jun 2025.

[33] World Medical Association . WMA Declaration of Helsinki – Ethical principles for medical research involving human participants. https://www.wma.net/policies‐post/wma‐declaration‐of‐helsinki‐ethical‐principles‐for‐medical‐research‐involving‐human‐subjects. Accessed 5 Jun 2025. 10.1001/jama.2024.21972 39425955

[34] Seiberle I , Währer J , Kron S , Flury K , Girardin M , Schocker A , et al. Collaborative swab performance comparison and the impact of sampling solution volumes on DNA recovery. Forensic Sci Int Genet. 2022;59:102716. 10.1016/j.fsigen.2022.102716 35512614

[35] Canfield JR , Jollie M , Worst T , Oechsle C . Comparison of swab types & elution buffers for collection and analysis of intact cells to aid in deconvolution of complex DNA mixtures. Forensic Sci Int. 2022;340:111448. 10.1016/j.forsciint.2022.111448 36087371

[36] Seratec® Gesellschaft für Biotechnologie mbH . Seratec® PSA Semiquant. 2019. https://www.seratec.com/docs/user_instructions/2019/IFU_PSA_DE_2019‐06.pdf. Accessed 5 Jun 2025.

[37] P.T.C. Laboratories . The erase sperm isolation kit. 2020. https://www.ptclabs.com/erase/. Accessed 5 Jun 2025.

[38] Promega Corporation . Maxwell® RSC instrument operating manual. TM411. Revised 12/21. Catalog Number AS4500. Madison, WI: Promega; 2021.

[39] Promega Corporation . PowerQuant® system technical manual #TMD047, Rev. 01/20. Madison, WI: Promega Corporation; 2020.

[40] Vandewoestyne M , Van Hoofstat D , Van Nieuwerburgh F , Deforce D . Automatic detection of spermatozoa for laser capture microdissection. Int J Leg Med. 2009;123:169–175. 10.1007/s00414-008-0271-1 18661142

[41] Sheth N , Duffy KR , Grgicak CM . High‐quality data from a forensically relevant single‐cell pipeline enabled by low PBS and proteinase K concentrations. J Forensic Sci. 2022;67:697–706. 10.1111/1556-4029.14956 34936089

[42] Sorg A , Gouy A , Tièche CC , Zieger M . Human background DNA on stones in an urban environment. Forensic Sci Int Genet. 2023;65:102880. 10.1016/j.fsigen.2023.102880 37116246

[43] Hansson O , Gill P . Characterisation of artefacts and drop‐in events using STR‐validator and single‐cell analysis. Forensic Sci Int Genet. 2017;30:57–65. 10.1016/j.fsigen.2017.04.015 28628901

[44] Team R . R: a language and environment for statistical computing. Vienna, Austria: R Foundation for Statistical Computing; 2021.

[45] Wickham H , François R , Henry L , Müller K . *Dplyr: a grammar of data manipulation*. R Package Version 107. 2021. https://CRAN.R‐project.org/package=dplyr. Accessed 5 Jun 2025.

[46] Kassambara A . *ggpubr: “ggplot2” based publication ready plots*. R package version 0.4.0. 2020. https://CRAN.R‐proje ct.org/package=ggpubr. Accessed 5 Jun 2025.

[47] Wickham H . ggplot2: elegant graphics for data analysis. New York, NY: Springer‐Verlag; 2016.

[48] Astrup BS , Thomsen JL , Lauritsen J , Ravn P . Detection of spermatozoa following consensual sexual intercourse. Forensic Sci Int. 2012;221:137–141. 10.1016/j.forsciint.2012.04.024 22607978

[49] Fonneløp AE , Johannessen H , Heen G , Molland K , Gill P . A retrospective study on the transfer, persistence and recovery of sperm and epithelial cells in samples collected in sexual assault casework. Forensic Sci Int Genet. 2019;43:102153. 10.1016/j.fsigen.2019.102153 31505370

[50] Hellerud BB , Bouzga M , Hoff‐Olsen P , Mevåg B . Semen detection: a retrospective overview from 2010. Forensic Sci Int Genet Suppl Ser. 2011;3(Suppl 2010):2010–2011. 10.1016/j.fsigss.2011.09.057

[51] Hall A , Ballantyne J . Novel Y‐STR typing strategies reveal the genetic profile of the semen donor in extended interval post‐coital cervicovaginal samples. Forensic Sci Int. 2003;136:58–72. 10.1016/S0379-0738(03)00258-5 12969621

[52] DiFrancesco J , Richards E . Persistence of spermatozoa: lessons learned from going to the sources. Sci Justice. 2018;58:244–247. 10.1016/j.scijus.2018.03.004 29685307

[53] Bahamondes L , Diaz J , Marchi NM , Castro S , Villarroel M , Macaluso M . Prostate‐specific antigen in vaginal fluid after exposure to known amounts of semen and after condom use: comparison of self‐collected and nurse‐collected samples. Hum Reprod. 2008;23:2444–2451. 10.1093/humrep/den283 18664473

[54] Mauck CK , Doncel GF . Biomarkers of semen in the vagina: applications in clinical trials of contraception and prevention of sexually transmitted pathogens including HIV. Contraception. 2007;75:407–419. 10.1016/j.contraception.2007.02.007 17519146

[55] Kulczycki A , Brill I , Snead MC , Macaluso M . Prostate‐specific antigen concentration in vaginal fluid after exposure to semen. Contraception. 2017;96:336–343. 10.1016/j.contraception.2017.07.004 28711645 PMC5737557

[56] Thurman A , Jacot T , Melendez J , Kimble T , Snead M , Jamshidi R , et al. Assessment of the vaginal residence time of biomarkers of semen exposure. Contraception. 2016;94:512–520. 10.1016/j.contraception.2016.05.012 27259675 PMC5075505

[57] Jamshidi R , Penman‐Aguilar A , Wiener J , Gallo MF , Zenilman JM , Melendez JH , et al. Detection of two biological markers of intercourse: prostate‐specific antigen and Y‐chromosomal DNA. Contraception. 2013;88:749–757. 10.1016/j.contraception.2013.08.003 24028752 PMC5845849

[58] Hochmeister MN , Budowle B , Rudin O , Gehrig C , Borer U , Thali M , et al. Evaluation of prostate‐specific antigen (PSA) membrane test assays for the forensic identification of seminal fluid. J Forensic Sci. 1999;44:1057–1060. 10.1520/jfs12042j 10486959

[59] Diamandis EP , Yu H . Nonprostatic sources of prostate‐specific antigen. Urol Clin North Am. 1997;24:275–282. 10.1016/S0094-0143(05)70373-6 9126224

[60] Lawson ML , Macaluso M , Bloom A , Hortin G , Hammond RK , Blackwell R . Objective markers of condom failure. Sex Transm Dis. 1998;25:427–432. 10.1097/00007435-199809000-00009 9773437

[61] Filella X , Molina R , Alcover J , Carretero P , Ballesta AM . Detection of nonprostatic PSA in serum and nonserum samples from women. Int J Cancer. 1996;68:424–427. 10.1002/(SICI)1097-0215(19961115)68:4<424::AID-IJC4>3.0.CO;2-2 8945610

[62] Macaluso M , Lawson L , Akers R , Valappil T , Hammond K , Blackwell R , et al. Prostate‐specific antigen in vaginal fluid as a biologic marker of condom failure. Contraception. 1999;59:195–201. 10.1016/s0010-7824(99)00013-x 10382083

[63] Denison SJ , Lopes EM , D'Costa L , Newman JC . Positive prostate‐specific antigen (PSA) results in semen‐free samples. Canadian Society of Forensic Science Journal. 2004;37(4):197–206. 10.1080/00085030.2004.10757576

[64] Kafarowski E , Dann K , Frappier JRH , Newman JC . Examination of semen‐free vaginal swabs for p30 using the SERATEC® PSA test kit: A further evaluation of the specificity of p30/PSA for semen identification. Proceedings of the MAAFS/MAFS/SAFS/CSFS Joint Meeting; 2004 Sept 19–24; Orlando, FL. Greencastle, PA: Mid‐Atlantic Association of Forensic Scientists; 2004.

[65] Allard JE , Davidson G , Baird A , Boyce M , Jones S , Lewis J , et al. The relevance of false positive acid phosphatase reactions indicative of the presence of seminal fluid from oral and vaginal samples. Sci Justice. 2023;63:477–484. 10.1016/j.scijus.2023.04.010 37453779

[66] Anslinger K , Bayer B . Whose blood is it? Application of DEPArray™ technology for the identification of individual/s who contributed blood to a mixed stain. Int J Leg Med. 2019;133:419–426. 10.1007/s00414-018-1912-7 30121738

[67] Vernarecci S , Ottaviani E , Agostino A , Mei E , Calandro L , Montagna P . Quantifiler® trio kit and forensic samples management: a matter of degradation. Forensic Sci Int Genet. 2015;16:77–85. 10.1016/j.fsigen.2014.12.005 25544252

[68] Elliott K , Hill DS , Lambert C , Burroughes TR , Gill P . Use of laser microdissection greatly improves the recovery of DNA from sperm on microscope slides. Int Congr Ser. 2004;1261:45–47. 10.1016/S0531-5131(03)01509-7 14550610

[69] Leppaluoto P . Vaginal flora and sperm survival. J Reprod Med. 1974;12:99–107. 4449105

[70] Randall B . Persistence of vaginal spermatozoa as assessed by routine cervicovaginal (PAP) smears. J Forensic Sci. 1987;32:678–683. 10.1520/JFS12372J 3598516

[71] Silverman EM , Silverman AG . Persistence of spermatozoa in lower genital tracts of women. JAMA. 1978;240:1875–1877. 567701 10.103/00006450-09000-00010

[72] Di Martino D , Giuffer G , Staiti N , Simone A , Le Donne M , Saravo L . Single sperm cell isolation by laser microdissection. Forensic Sci Int. 2004;146:151–153. 10.1016/j.forsciint.2004.09.046 15639564

[73] Francolini M , Lavitrano M , Lamia CL , French D , Frati L , Cotelli F , et al. Evidence for nuclear internalization of exogenous DNA into mammalian sperm cells. Mol Reprod Dev. 1993;34:133–139. 10.1002/mrd.1080340204 8442952

[74] Spadafora C . Sperm cells and foreign DNA: a controversial relation. Bioessays. 1998;20:955–964. 10.1002/(SICI)1521-1878(199811)20:11<955::AID-BIES11>3.0.CO;2-8 9872062

[75] Hennekens CM , Cooper ES , Cotton RW , Grgicak CM . The effects of differential extraction conditions on the premature lysis of spermatozoa. J Forensic Sci. 2013;58(3):744–752. 10.1111/1556-4029.12098 23550664

[76] Tereba BA , Flanagan L , Mandrekar P , Olson R . DIFFEREX™ SYSTEM: a new, rapid method to separate sperm and epithelial cells. Profiles in DNA. 2004;20:8–10.

[77] Greenspoon SA , Scarpetta MA , Drayton ML , Turek SA . QIAamp spin columns as a method of DNA isolation for forensic casework. J Forensic Sci. 1998;43:1024–1030. 10.1520/jfs14351j 9729819

[78] Vandewoestyne M , Van Hoofstat D , Van Nieuwerburgh F , Deforce D . Suspension fluorescence in situ hybridization (S‐FISH) combined with automatic detection and laser microdissection for STR profiling of male cells in male/female mixtures. Int J Leg Med. 2009;123:441–447. 10.1007/s00414-009-0341-z PMC2754505 19319556

[79] Golomingi R , Haas C , Dobay A , Kottner S , Ebert L . Sperm hunting on optical microscope slides for forensic analysis with deep convolutional networks – a feasibility study. Forensic Sci Int Genet. 2022;56:102602. 10.1016/j.fsigen.2021.102602 34700216

