# Supplementary material for: Evaluating novel and conventional cell‐separation techniques for sexual assault investigations
Source: J Forensic Sci. 2025 Jul 11;70(5):1704–20. doi: 10.1111/1556-4029.70131 (PMC12424101; doi:10.1111/1556-4029.70131)
Supplement: Supplementary file 1 — Data S1. [file JFO-70-1704-s001.docx]

**Supplemental Information**


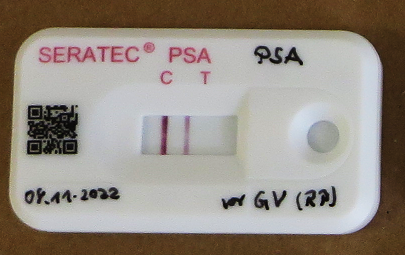

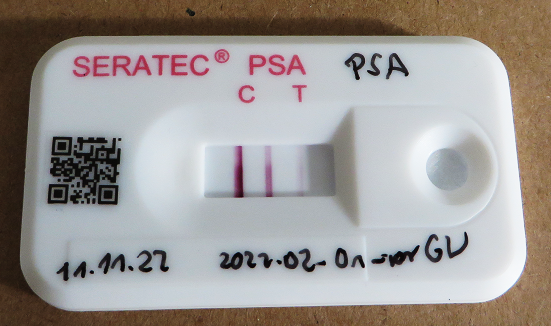

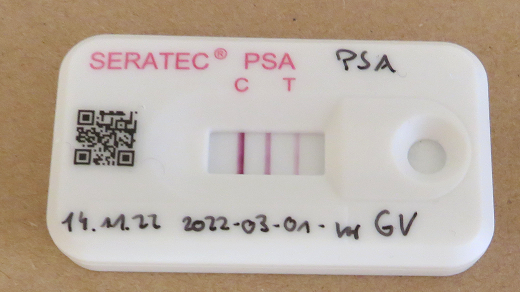

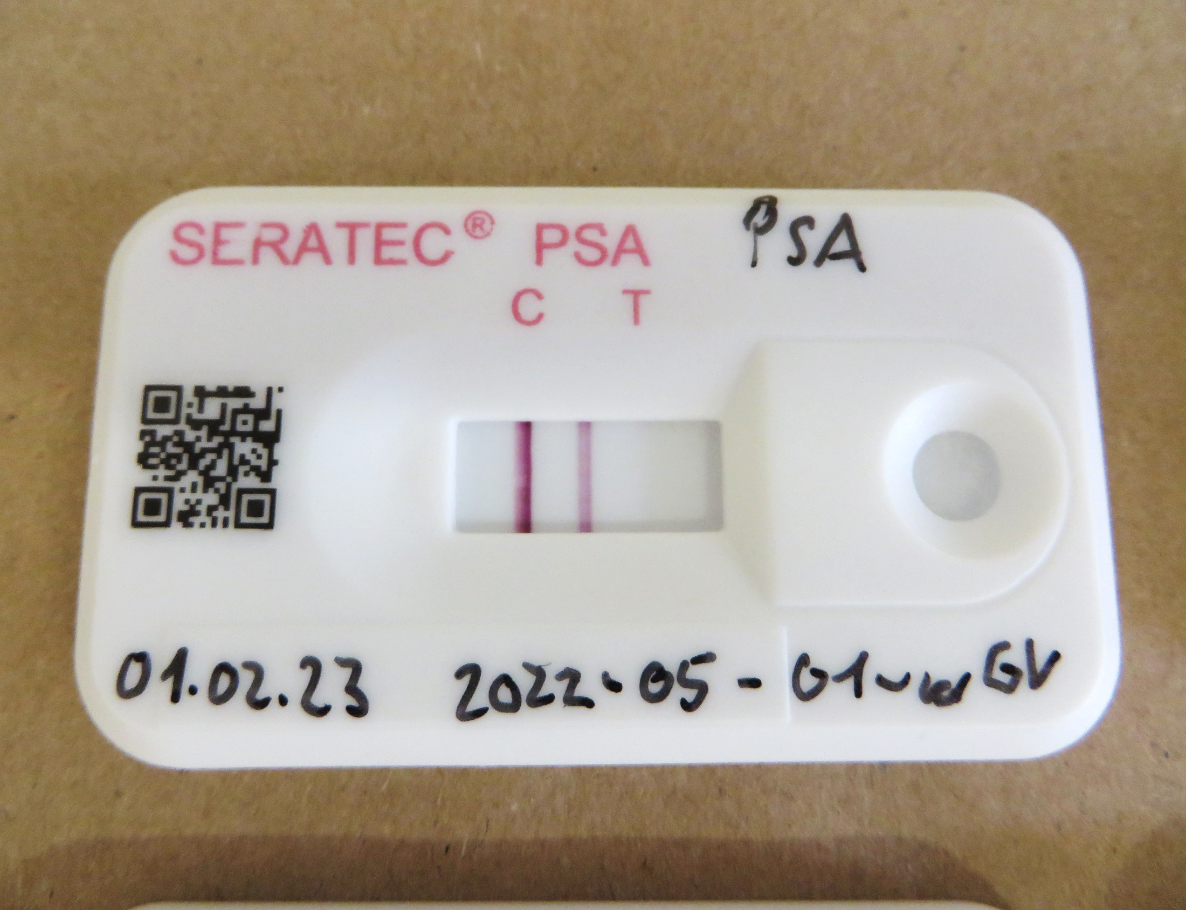

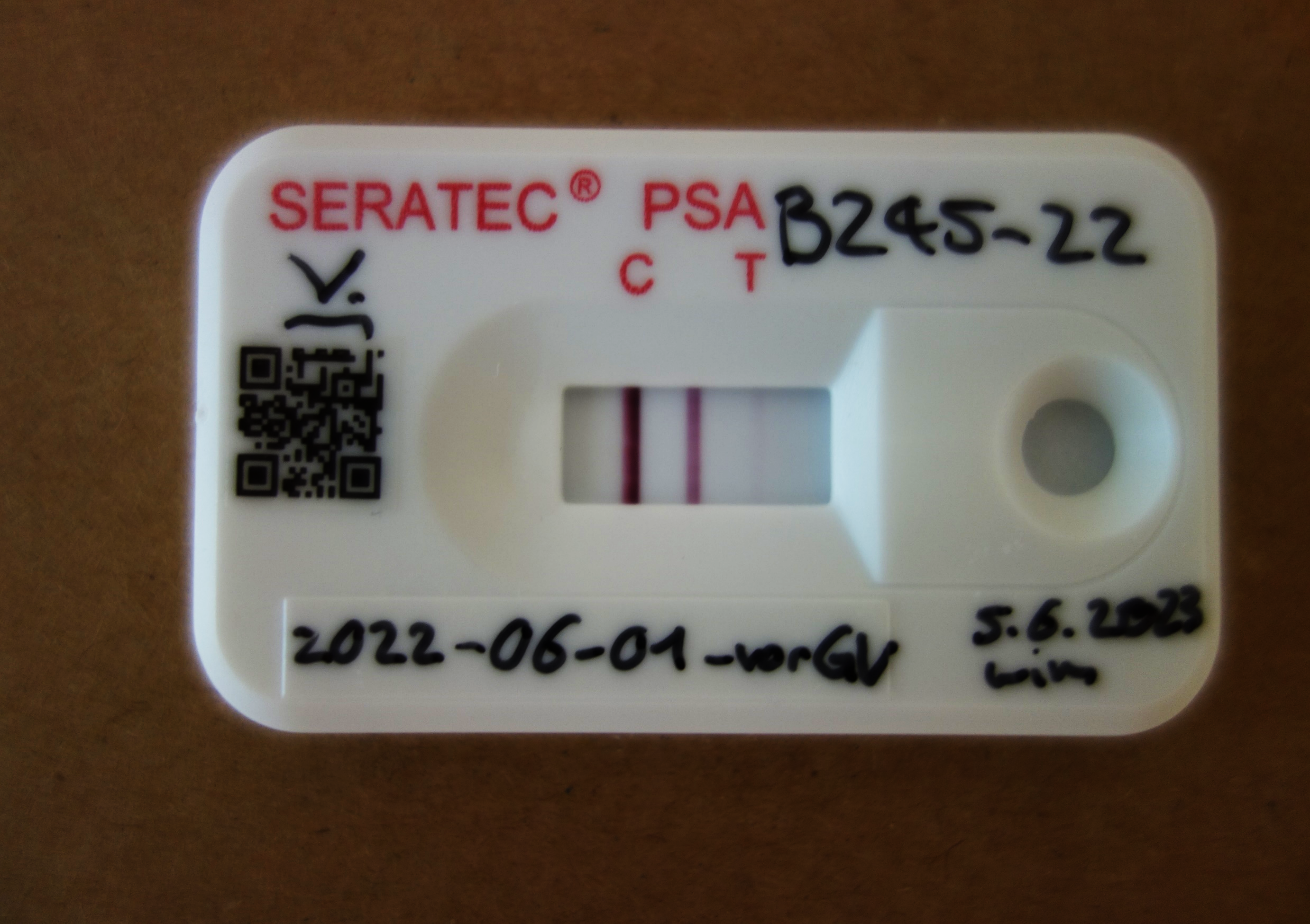

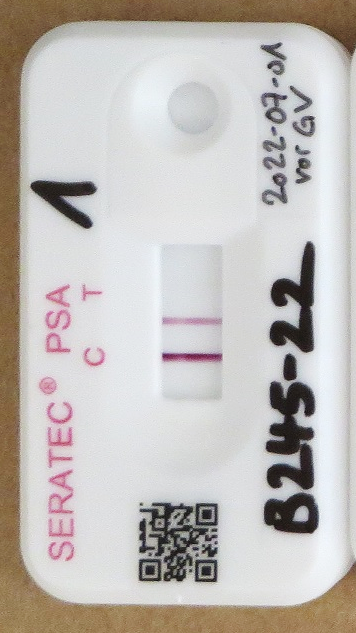

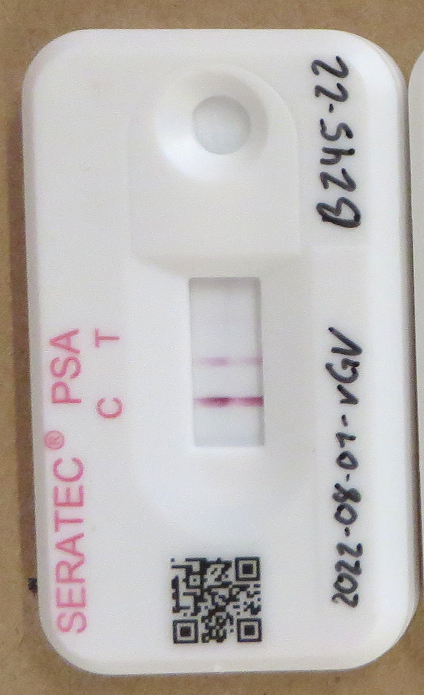

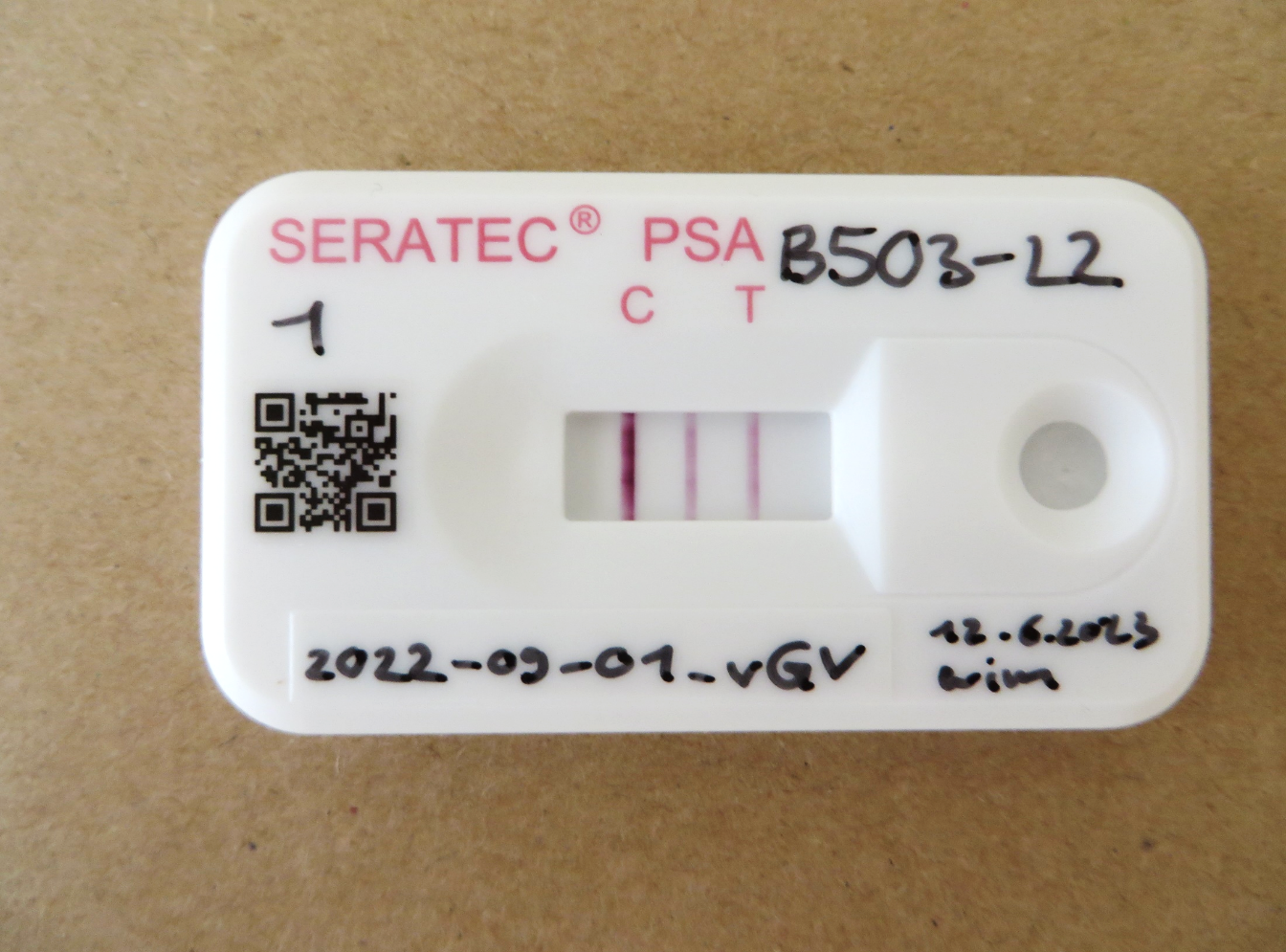

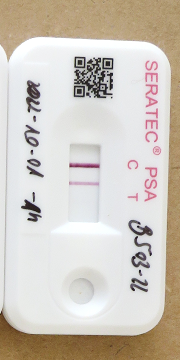

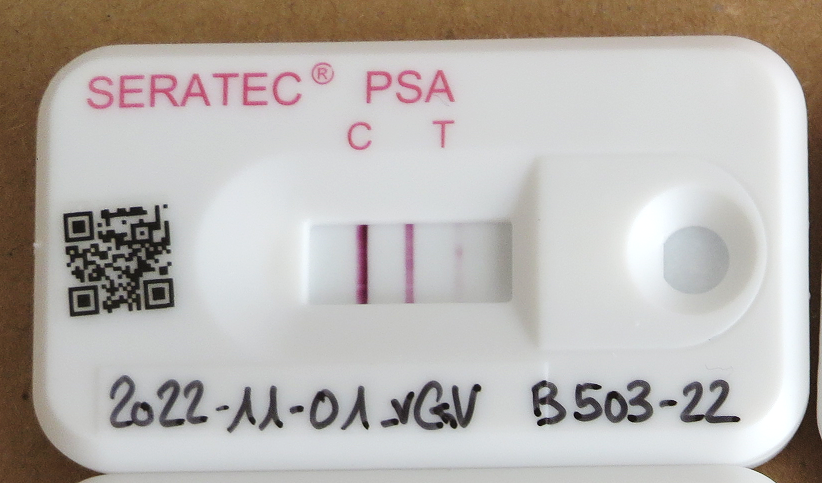


2022-01-01

2022-02-01

2022-03-01

2022-04-01

2022-05-01

2022-06-01

2022-07-01

2022-08-01

2022-09-01

2022-10-01

FIGURE S1 Baseline samples. Prostate-specific antigen (PSA) test results of baseline samples (i.e., sample collected prior sexual intercourse after one week of abstinence) from all study participants. The positive test lines are framed.

*
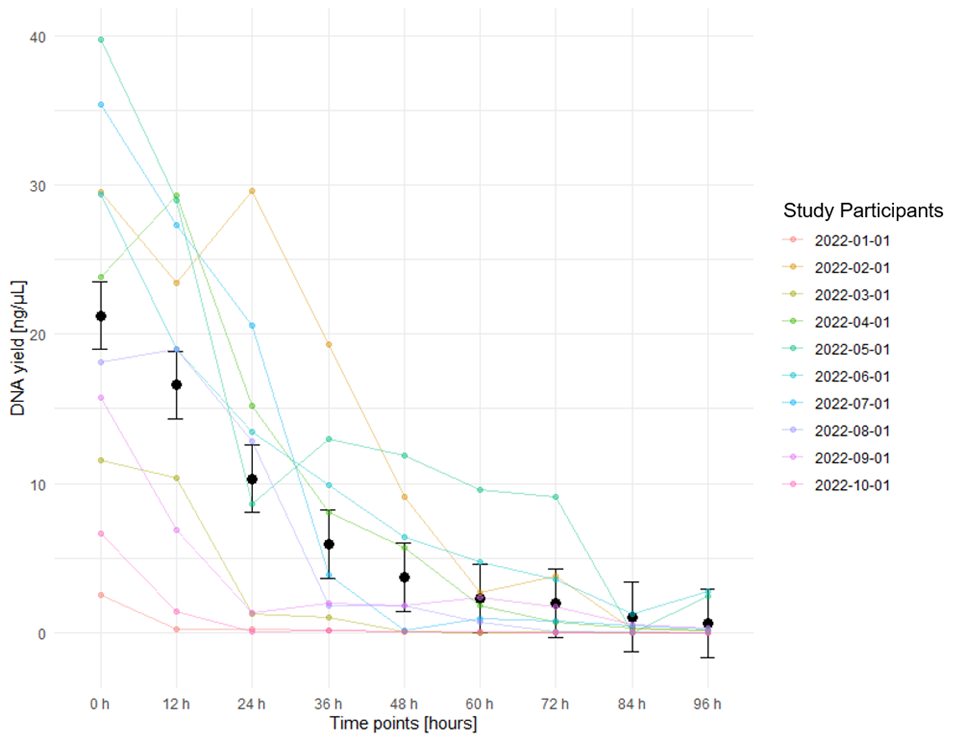
*

FIGURE S2 Y-chromosomal DNA. Line and scatter plot summarizing the relationship between male DNA yield (ng/µL) and TSI (hours) in the sperm fraction. Error bars depict the SE (standard error of the mean).


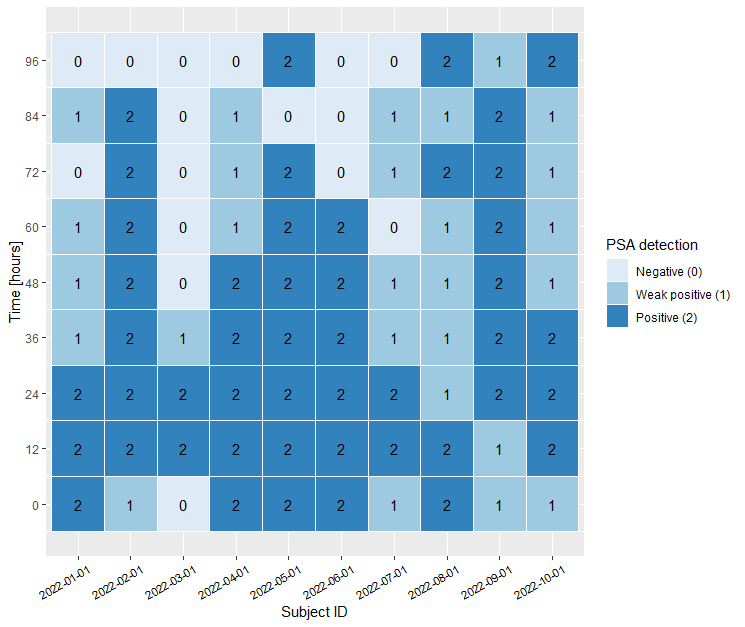


FIGURE S3 PSA-Test results. Heat map depicting positive (2), weak positive (1) and negative (0) PSA test results of all ten participants over a period of 96 hours.

TABLE S1 Randomized sample assignment per study cohort.

| **Study cohort** | **Swab 1** | **Swab 2** | **Swab 3** |
| --- | --- | --- | --- |
| **2022-01-01** | DE | DEPArray™ | LCM |
| **2022-02-01** | LCM | DE | DEPArray™ |
| **2022-03-01** | DEPArray™ | LCM | DE |
| **2022-04-01** | LCM | DE | DEPArray™ |
| **2022-05-01** | DEPArray™ | LCM | DE |
| **2022-06-01** | DE | DEPArray™ | LCM |
| **2022-07-01** | LCM | DE | DEPArray™ |
| **2022-08-01** | DEPArray™ | LCM | DE |
| **2022-09-01** | DE | DEPArray™ | LCM |
| **2022-10-01** | LCM | DE | DEPArray™ |

TABLE S2 Total allele counts as well as the amount of shared alleles (excluding Amelogenin) according to the reference profile obtained from buccal swabs processed per study participant.

| Study cohort | # Allele counts (Female) | # Allele counts (Male) | # Shared alleles |
| --- | --- | --- | --- |
| 2022-01-01 | 28 | 30 | 12 |
| 2022-02-01 | 29 | 30 | 8 |
| 2022-03-01 | 31 | 28 | 8 |
| 2022-04-01 | 27 | 31 | 8 |
| 2022-05-01 | 28 | 26 | 9 |
| 2022-06-01 | 24 | 29 | 11 |
| 2022-07-01 | 31 | 30 | 11 |
| 2022-08-01 | 28 | 28 | 8 |
| 2022-09-01 | 29 | 29 | 7 |
| 2022-10-01 | 30 | 30 | 6 |

TABLE S3 Total number of showers and their respective time points (time since intercourse (TSI)) per female study participant.

| **Study cohort** | **# Showers** | **TSI** |
| --- | --- | --- |
| **2022-01-01** | NA^*^ | NA^*^ |
| **2022-02-01** | 2 | 13 and 61 hours |
| **2022-03-01** | 3 | 18, 48, and 84 hours |
| **2022-04-01** | 3 | 5, 54, and 94 hours |
| **2022-05-01** | 4 | 3, 24, 48, and 72 hours |
| **2022-06-01** | 4 | 0, 27, 43, 75 hours |
| **2022-07-01** | 1 | 48 hours |
| **2022-08-01** | 5 | 1, 12, 32, 62, 85 hours |
| **2022-09-01** | 4 | 0, 23, 50, 79 hours |
| **2022-10-01** | 2 | 15, 87 hours |

*Showering was not accurately documented.

TABLE S4 Summary of the technical data of differential extraction, LCM and DEPArray™.

| Feature | Differential Extraction | LCM | DEPArray™ |
| --- | --- | --- | --- |
| Type of separation | Cell enrichment | Cell capture | Cell capture |
| Sample composition | Heterogeneous mixtures  (Non-Sperm:Sperm)  Cell-free DNA embedded | Heterogeneous mixtures  (Non-Sperm:Sperm)  Intact cells; loss of cell-free DNA | Heterogeneous/homogenous mixtures (blood, epithelial, sperm)  Intact cells; loss of cell-free DNA |
| Mixture complexity | 2-person mixture | ≥ 2-person mixture | ≥ 2-person mixture |
| Sample load | Entire sample | 2-6 µL/slide | 6,000 cells/run |
| Sample amount needed | No studies | No studies | No studies |
| Sample loss | Yes, during separation and extraction steps [11]. | Yes, cell rupture (laser energy) | Yes, during sample load and routing + non-retrieval of non-routable cells |
| Sperm detection | e.g., additional immune-based presumptive test and microscopy | Image-based | Image-based |
| Risk of contamination | Possible | Unlikely | Unlikely |
| Disposals | Typical consumables | Typical consumables  (Membrane slides) | Typical consumables  Single-use cartridge |
| Time | 1-2 day(s) | 2-3 day(s) | 3-5 days |
| Automation | Manual (automatic devices available) | Manual (automatic sperm detection available) | Semi-automatic |
| Training | Required  Effort medium | Required  Effort medium | Required  Effort high |
| Application | Restricted to sexual assaults | Mainly sexual assaults | Not limited |
